# Developing a New Approach Methodology Framework to Assess Biological Responses to Nanoplastics: Insights from Polystyrene and Biodegradable Particles

**DOI:** 10.3390/nano16110668

**Published:** 2026-05-26

**Authors:** Chiara Ritarossi, Simonetta Palleschi, Maria Condello, Barbara Rossi, Luca Pannone, Chiara Laura Battistelli, Cecilia Bossa, Giovanni Libralato, Simone Martinelli, Cristina Andreoli

**Affiliations:** 1 Environment and Health Department, Istituto Superiore di Sanità, 00161 Rome, Italy; chiara.ritarossi@unina.it (C.R.); simonetta.palleschi@iss.it (S.P.); barbara.rossi@iss.it (B.R.); chiara.battistelli@iss.it (C.L.B.); cecilia.bossa@iss.it (C.B.); 2 Department of Biology, University of Naples Federico II, Via Cinthia 26, 80126 Naples, Italy; giovanni.libralato@unina.it; 3 National Center for Drug Research and Evaluation, Istituto Superiore di Sanità, 00161 Rome, Italy; maria.condello@iss.it; 4 Department of Oncology and Molecular Medicine, Istituto Superiore di Sanità, 00161 Rome, Italy; luca.pannone@iss.it (L.P.); simone.martinelli@iss.it (S.M.)

**Keywords:** microplastics, nanoplastics, biodegradable plastics, polystyrene nanoplastics, polycaprolactone nanoplastics, in vitro intestinal barrier model, *C. elegans*, NAMs, genotoxicity, barrier integrity, oxidative stress

## Abstract

The widespread presence of micro- and nanoplastics (MNPs) in the environment represents an emerging risk for human and environment health. New Approach Methodologies (NAMs) offer valuable tools to improve the mechanistic understanding of nanoscale processes and support hazard identification without animal testing. This study investigated the biological effects of exposure to 0–100 µg/mL 100 and 20 nm polystyrene (PS-NPs) and 100 nm polycaprolactone nanoplastics (PCL-NPs) using advanced in vitro intestinal models and the 3R-compliant in vivo *Caenorhabditis elegans* model. In vitro endpoints included cytotoxicity, oxidative stress, DNA damage, cellular internalization, and barrier integrity, while in vivo analyses focused on oxidative stress and locomotor behavior across multiple exposed generations. PS-NPs induced significant DNA damage in vitro, particularly at ≥50 µg/mL after 24–48 h exposure, and were rapidly internalized by cells, with 20 nm particles also detected in the nucleus. In contrast, 100 nm PCL-NPs elicited weaker biological responses. In vivo, PS-NPs caused an increase in oxidative stress response and locomotor behavior across exposed generations, whereas PCL-NPs produced milder effects, consistent with in vitro findings. These results support the potential of integrated NAMs for assessing human health risks associated with MNP exposure within a One Health framework.

## 1. Introduction

Micro- and nanoplastics (MNPs) are increasingly recognized as emerging contaminants of concern due to their widespread presence in food, water, and the environment, resulting in continuous human exposure. These particles, originating from the degradation of larger plastics or direct release from consumer products and industrial sources, span sizes from the millimetre to the nanometre scale. The predominant exposure routes are ingestion (via food, drinking water, and dust), inhalation (via indoor and outdoor air), and to a lesser extent dermal contact (especially for the smallest particles) [1].

Globally, approximately 8–12 million metric tonnes of plastic waste enter the oceans each year, leading to MNP contamination in aquatic environments and their transfer across trophic levels [2]. This is also relevant for human health, as MNPs may reach humans through dietary exposure. However, quantitative data on human intake remain limited, and experimental studies, including the present one, often use relatively high concentrations and acute exposure scenarios to investigate potential toxicological mechanisms [2].

Among the different human exposure routes, oral intake is considered particularly relevant, making the gastrointestinal tract a primary site of interaction between MNPs and biological systems. Due to their small size, MNPs may be bioavailable and interact with intestinal cells, potentially impairing epithelial barrier integrity and inducing adverse effects through mechanisms such as oxidative stress, inflammation, and genotoxicity [3]. Despite growing experimental evidence, the hazard identification of MNPs remains fragmented and insufficiently standardized to effectively support regulatory risk assessment [4].

The complexity of nanoparticle exposure, encompassing a wide range of particle sizes, polymer types, surface properties, and transformation processes, challenges traditional toxicological approaches, which are often time-consuming, ethically challenging, and limited in human relevance. In response, regulatory bodies such as OECD, EFSA, and ECHA increasingly promote the use of New Approach Methodologies (NAMs). NAMs encompass a variety of testing strategies, including in vitro and invertebrate in vivo models, high-content screening, omics technologies, and computational tools, designed to provide mechanistic insights and improve human health relevance. NAMs also adhere to the 3Rs principles (Replacement, Reduction, and Refinement of animal use), while maintaining or enhancing the predictive power of toxicity assessments [5]. However, the application of NAMs to MNPs is still hindered by the lack of harmonized testing strategies, standardized material characterization, and comparable datasets, all of which are critical for regulatory acceptance and integration into risk assessment frameworks [6].

Within this context, the selection of representative model materials is a critical aspect of study design. In the present work, polystyrene (PS-NPs) and polycaprolactone (PCL-NPs) nanoplastics were selected as model systems to enable a comparative hazard evaluation across polymer types. PS is one of the most extensively used reference materials in NPs research due to its commercial availability, physicochemical stability, and widespread use in toxicological studies, which facilitates data comparability across studies [7]. PCL is a biodegradable polyester widely used in biomedical and industrial applications. Although generally considered biocompatible, its slow degradation may lead to persistence and the release of oligomeric products, raising questions about its biological interactions at the micro and nanoscale [8]. Emerging evidence suggests that PCL MNPs can interact with epithelial systems and induce biological responses, although available data remain limited and sometimes inconsistent, highlighting the need for more standardized experimental approaches to improve the reliability, comparability, and scientific robustness of toxicity studies, as recommended by several authors [9]. Despite not being among the most produced biodegradable polymers, PCL’s well-characterized physicochemical properties make it a suitable model for investigating potential biological responses at the nanoscale. This study approach is not intended to reflect production volumes or environmental prevalence, but rather to provide a controlled framework to investigate polymer dependent effects within a tiered testing strategy.

Biodegradable plastics have been proposed as sustainable alternatives to conventional polymers to mitigate environmental persistence and associated risks [10]. However, current evidence on their biological effects, particularly at the micro- and nanoscale and following oral exposure, remains limited and sometimes inconsistent. Moreover, biological responses to NPs are not restricted to epithelial cells, but may also involve the activation of immune and inflammatory pathways, which are increasingly recognized as key determinants of toxicity [11]. Therefore, although the assessment of immunotoxicological endpoints was beyond the scope of the present study, their integration of such endpoints represents an important perspective for future NAM-based testing strategies.

In this study, an integrated tiered NAM-based approach combining complementary in vitro and in vivo models was applied to evaluate the hazard profiles of 20 and 100 nm PS-NPs and 100 nm PCL-NPs. The approach combines human intestinal in vitro models, including Caco-2 monolayers and an advanced Caco-2/HT29-MTX/Raji-B co-culture system that recapitulates key structural and functional features of the intestinal barrier, such as mucus production and M-cell-mediated translocation [12], with the in vivo alternative model *Caenorhabditis elegans*, compliant with the 3Rs principles [5,13,14]. A panel of biologically relevant endpoints, including cytotoxicity, genotoxicity, oxidative stress, barrier integrity, nanoparticle internalization, and organism-level functional effects, was assessed to support hazard identification and enable comparison across polymer types and particle sizes.

The proposed approach should be considered a proof-of-concept for an integrated NAM-based tiered testing strategy, in which complementary endpoints and model systems are combined to explore different levels of biological organization, from cellular responses to organism-level effects, thereby supporting a more structured interpretation of the data. By integrating complementary techniques, NAMs may contribute to the generation of mechanistically informative datasets that, if progressively developed in accordance with the FAIR (findable, accessible, interoperable, and reusable) principles [15], could support scientific exchange and future data integration efforts. In this context, greater methodological harmonization may help improve the comparability and transparency of results across studies, potentially contributing to evidence-based evaluation approaches. This aspect is particularly relevant in nanotoxicology, where variability in material characterization and testing conditions often limits data comparability [16,17].

Overall, this work aims to illustrate the potential of a NAM-based approach to generate mechanistically informative data and to support the development of more integrated strategies for MNP hazard assessment.

## 2. Materials and Methods

### 2.1. Suspensions Preparation and Particle Characterization

Spherical 100 nm PS-NPs (catalog number: Latex Beads LB1 MFCD00131491) and PCL-NPs (catalog number: PCL100-5ML-A) were obtained from Merck/Sigma-Aldrich (St. Louis, MO, USA) The 20 nm PS-NPs (catalog number: Polymer Microsphere Suspension 3020A) were obtained from Thermo Fisher Scientific (Waltham, MA, USA).

The NP suspensions were prepared and characterized following the Nanogenotox Standard Operative Procedure (SOP) https://www.anses.fr/en/system/files/nanogenotox_deliverable_6.pdf (accessed on 10 March 2026) [18]. The NPs were first diluted in ddH_2_O to a working concentration of 5000 µg/mL and the final concentrations were then obtained by dilutions in cell culture medium. Particle sizes and zeta-potential (ζ-potential) were verified by Dynamic Light Scattering (DLS) with a Zetasizer Nano ZS (Malvern Panalytical GmbH, Kassel, Germany). Details of the procedure are described in Supplementary Materials.

### 2.2. Cell Culture and Treatment

For the in vitro experiments, a monoculture model of intestinal epithelial cells (Caco-2, HTB-37 clone, a colorectal adenocarcinoma cell line), and an intestinal barrier model composed of Caco-2, HT29-MTX E12 cells (a mucus-secreting subclone of the human colorectal adenocarcinoma HT29 cell line), in a 9:1 ratio, and Raji-B (a lymphoblastoid cell line derived from Burkitt’s lymphoma), have been used.

All cell lines were obtained from the American Type Culture Collection (ATCC, Manassas, VA, USA).

The establishment and differentiation of the Caco-2/HT29-MTX/Raji-B in vitro barrier model was performed as described in Vincentini et al. 2022 [12]. Detailed cell culture conditions and triculture establishment procedures are provided in the Supplementary Materials.

For monoculture experiments, 250,000 Caco-2 cells were seeded in 12-well cell culture plates for three days and allowed to reach confluence prior to treatment.

Unless otherwise specified, for in vitro treatments, cells were exposed to different concentrations (0, 25, 50, and 100 µg/mL) of freshly prepared NP suspensions for up to 48 h and then analysed for the selected endpoint(s). For each condition, at least two independent experiments, each in duplicate, were performed. At the time of treatment, the cell culture medium was replaced with medium in which the NPs had been freshly suspended. For barrier experiments, medium containing NPs was added to the apical compartment, while the basolateral compartment contained NP-free medium only. The concentration range (0–100 µg/mL) was selected based on preliminary cytotoxicity assessments, ensuring coverage of sub-cytotoxic conditions suitable for mechanistic evaluation. In addition, this range is consistent with recommendations from regulatory agencies and international guidelines, which indicate 100 µg/mL as the upper limit for testing nanomaterials in vitro to avoid artefacts and interactions with assay components and ensure data reliability [18].

### 2.3. Cytotoxicity

The NPs cytotoxicity was assessed using the 3-(4,5-dimethylthiazol-2-yl)-5-(3-carboxymethoxyphenyl)-2-(4-sulfophenyl)-2H-tetrazolium (MTS) cell viability assay, through the CellTiter 96^®^ AQueous One Solution Cell Proliferation Assay (Promega Corporation, Madison, WI, USA). It was performed according to the SOP developed within the EU NanoValid project [19] with minor modifications to adapt the procedure for Caco-2 cells as implemented in the NANoREG project. The MTS assay measures cellular metabolic activity as an indicator of cell viability and cytotoxicity, based on the mitochondrial enzymatic reduction of the MTS tetrazolium compound to a soluble formazan product in metabolically active cells. Briefly, Caco-2 cells were cultured into 96-well culture plates. After 24 h, cells were treated with five different NPs concentrations of 0, 1, 10, 25, 50, and 100 μg/mL for 24 and 48 h. Benzalkonium chloride (BC) (Merck/Sigma-Aldrich, St. Louis, MO, USA) was used as a positive control (0–50 μg/mL). Detailed information about the assay procedure is provided in Supplementary Materials. The results were reported and quantified using the NanoValid template. For each NP analysed, at least two independent experiments were performed for each exposure time, each including three technical replicates. In addition, blank wells (without cells) containing all tested NP concentrations were included in duplicate to assess potential assay interference. In addition, cell viability was assessed at the time of cell harvesting using the Trypan Blue (Merck/Sigma-Aldrich, St. Louis, MO, USA) exclusion assay.

### 2.4. Transmission Electron Microscopy

After treatment with PS-NPs of 20 and 100 nm (100 μg/mL) for 1, 4, and 24 h, detached and adherent cells were collected. After centrifugation, samples were fixed with 2.5% glutaraldehyde (Merck/Sigma-Aldrich, St. Louis, MO, USA) in 0.1 M cacodylate buffer (Merck/Sigma-Aldrich, St. Louis, MO, USA) (pH 7.3) at room temperature for 20 min. They were postfixed with 1% OsO_4_ in 0.2 M cacodylate buffer (pH 7.3) at room temperature for 30 min; then, they were dehydrated with ascending concentrations of ethanol (Merck/Sigma-Aldrich, St. Louis, MO, USA) and embedded in epoxy resin (TAAB Laboratories Equipment Limited, Aldermarton, UK). Ultrathin sections were obtained with an LKB Ultratome Nova ultramicrotome (LKB, Bromma, Sweden), and then they were examined with a Philips EM208S transmission electron microscope (FEI Company, Eindhoven, The Netherland). The images in the figures are representative of three fields observed for each sample. They were processed with the Adobe Photoshop 2026 program. The measurements could not be performed for PCL-NPs, as the material is no longer available from the manufacturer.

### 2.5. In Vitro Oxidative Stress

Oxidative stress in Caco-2 monolayers was evaluated by measuring intracellular levels of reduced (rGSHi) and total glutathione (tGSHi) and extracellular levels of cysteine (tCYSe), cysteinylglycine (tCYSGLYe), homocysteine (tHCYe) and glutathione (tGSHe). These low-molecular-weight thiols are involved in glutathione metabolism and cellular redox responses and have been reported as indicators of oxidative or electrophilic stress in vitro [20,21,22]. This approach provides a cumulative measure of the cellular response to oxidative insult while avoiding potential NP interference in commonly used fluorometric intracellular ROS assays. rGSHi was measured photometrically by its reaction with 5,5′-dithiobis (2-nitrobenzoic acid) [20]. tGSHi, tGSHe, tCYSe, tCYSGLYe, and tHCYe were determined by HPLC with fluorescence detection, according to protocols previously established in our laboratory [23], with minor modifications. Protein concentration in cell lysates was quantified by the Bradford assay. Full details of the experimental procedure are reported in the Supplementary Materials. Cellular glutathione levels were expressed as nmol of total glutathione per mg of protein and as the percentage ratio of rGSHi to tGSHi. Thiol concentrations in culture media were expressed as µmol/L. Two independent experiments were performed for each type of NP, each including two technical replicates per conditions.

### 2.6. Comet Assay

DNA damage, as double and single strand breaks, was evaluated by alkaline comet assay. After 96 h from seeding, cells were exposed to the selected concentrations of NPs and 150 μM of H_2_O_2_ (Merck/Sigma-Aldrich, St. Louis, MO, USA) as positive control. At the end of treatment, cell viability was checked using Trypan Blue exclusion assay, and the comet assay was performed according to the protocol described in Andreoli and collaborators 1997 [24].

The whole assay procedure has been described in Supplementary Materials. The FPG (formamidopyrimidine-DNA glycosylase) (Merck/Sigma-Aldrich, St. Louis, MO, USA) modified version of comet assay, to detect oxidized DNA bases, was performed according to the protocol developed by Comet Assay Interest Group [25]. The percentage of DNA in the comet tail was selected as the most suitable parameter for evaluating DNA damage and quantified using an image analysis system (IAS 2000 Delta Sistemi Srl, Bergamo, Italy). Three independent experiments were performed for each PS-NP size, each including two technical replicates, while two independent experiments, due to limited availability of materials from the supplier, were conducted for PCL-NPs under the same conditions. For each treatment condition, at least 100 cells were analyzed using the image analysis system.

### 2.7. Paracellular Permeability

The paracellular transport of Lucifer yellow CH di-lithium salt (LY) 457 D (Merck/Sigma-Aldrich, St. Louis, MO, USA) was evaluated on the tri-culture intestinal barrier model on 3 μm pore size Millipore inserts, placed in 12-well plates, to assess the barrier’s permeability after the exposure to NPs. Before conducting the experiments, membrane integrity was verified by transepithelial electrical resistance (TEER) measurements, and the paracellular permeability was evaluated only on inserts exhibiting acceptable TEER values (≥150 Ω·cm^2^). Three independent experiments were performed for each PS-NP size, each including two technical replicates, while two independent experiments, due to limited availability of materials from the supplier, were conducted for PCL-NPs under the same conditions, including a total of 12 inserts per experiment for both exposure times (24 and 48 h). Additional information about the assay procedure is provided in Supplementary Materials.

### 2.8. C. elegans Strains and Culture

The strains Bristol N2 (wild-type) and CL2166 (dvIs19 [(pAF15)gst-4p::GFP::NLS] were obtained from the Caenorhabditis Genetics Center (University of Minnesota, Minneapolis, MI, USA). Strains were grown on nematode growth medium (NGM) agar plates at 20 °C, seeded with *E. coli* OP50 bacteria as a food source.

#### 2.8.1. *C. elegans* Exposure Study Design

The exposure scenario to NPs was designed to evaluate the effects of nematodes’ prolonged exposure. PS-NPs concentrations (0.1–100 µg/mL) were selected based on a previous study [26], which demonstrated that 10, 50, and 100 µg/mL PS-NPs induce toxicity in *C. elegans*. Moreover, exposure was continuously applied across all generations (P0–F2), ensuring that each generation was directly subjected to the same treatment conditions. Additional details regarding the exposure scenario are provided in the Supplementary Materials.

#### 2.8.2. Behavioural Assays in *C. elegans*

Locomotion parameters were analysed quantitatively using a custom-made automated tracking system, which has been previously described [27]. Two endpoints (head thrashes in M9 liquid culture and body bends on agar plates) were selected to assess nematode locomotion. A head thrash is defined as a change in bending direction at the body mid-region, whereas a body bend is defined as a change in the direction of the posterior bulb of the pharynx along the y axis when the worm is traveling along the x axis [28]. Two independent experiments were performed for each NP. Twenty nematodes were analysed per experimental group. Embryonic and larval lethality were investigated as previously described [29].

#### 2.8.3. Oxidative Stress Response in *C. elegans*

The transgenic *C. elegans* strain CL2166 (dvIs19 [(pAF15) gst-4p::GFP::NLS]) was used to assess activation of the cellular oxidative stress response. In this strain, GFP expression is driven by the gst-4 promoter and serves as a reporter of *skn-1*-dependent transcriptional activation in response to oxidative stress. Following exposure, nematodes were gently transferred into microscope slides prepared with 2% agarose pads containing 10 mM sodium azide as an anaesthetic. GFP fluorescence was observed using an inverted fluorescence microscope (Nikon Eclipse Ti2-E equipped with DIC optics; Nikon Corporation, Tokyo, Japan). Two independent experiments were performed for each NP. Twenty nematodes were analysed per experimental group.

### 2.9. Statistical Analyses

For in vitro experiments, treatment effects were analysed using two-way ANOVA with time and concentration as factors. When a significant effect was observed, multiple comparisons were performed using Dunnett’s post hoc test to compare each treated group with the corresponding control. For *C. elegans* experiments, differences between control and treated groups were evaluated using one-way ANOVA followed by Dunnett’s post hoc test; comparison between 100 and 20 nm PS-NPs were performed using Bonferroni’s post hoc test for multiple comparisons.

All analyses were performed using GraphPad Prism (v10.1.1), and statistical significance was set at *p* < 0.05.

### 2.10. FAIRification Process

In this initial phase, following the generation of project data, the focus shifts to FAIRification, the process of transforming data to comply with the FAIR principles. This involves evaluating the feasibility of using and adapting the available templates to the specific needs of the project. The primary goal is to generate as much FAIR data as possible to optimize its future reuse across various scenarios, particularly for risk assessment and regulatory purposes. This includes identifying missing endpoints and developing new templates through collaboration between data providers and domain experts.

As part of the ongoing FAIRification process, we evaluated the templates available on the NanoSafety Data Interface (Nanosafety data interface, https://enanomapper.adma.ai/, accessed on 10 February 2026) to confirm their suitability for the project’s key endpoints. These templates were extracted from the Template Wizard, an online form developed within the eNanoMapper interface. The Template Wizard was designed specifically to facilitate the capture of experimental data and metadata via community-agreed templates, ensuring that different data types remain linked. By hyperlinking SOPs and workflows directly to the templates, the system further enhances data harmonization and interoperability [30].

## 3. Results

The results are presented following a tiered NAM-based approach, from in vitro intestinal models to a complementary in vivo 3R-compliant model, in order to evaluate the consistency and biological relevance of NP-induced effects across different levels of complexity.

### 3.1. Nanoplastic Particles Characterization

Table 1 summarizes the properties of the particles used in this study, as provided by the manufacturer. DLS analysis showed that the hydrodynamic sizes of the NPs suspended in water were consistent with the expected values, and that PS-NPs exhibited a strongly negative ζ-potential, whereas PCL-NPs showed near-neutral values (Table 2). After dispersion in cell culture medium, the size of PS-NPs increased and their ζ-potential decreased in magnitude, suggesting the immediate formation of a protein corona. Over time, the size of 100 nm PS-NPs, but not 20 nm PS-NPs, showed a slight increase (up to 25% after 48 h), indicating minor agglomeration of the larger PS particles throughout the experiments.

For PCL-NPs, it is important to note that the refractive index of PCL is close to that of serum proteins, leading to partial overlap between nanoparticle- and endogenous, medium-derived scattering populations. Consequently, the intensity-weighted size distribution of 100 nm PCL-NPs in cell culture medium was highly polydisperse, preventing reliable determination of the Z-average. Therefore, the hydrodynamic size of PCL-NPs was evaluated based on the main peak (Peak 1) of the intensity distribution.

In contrast to PS-NPs, PCL-NPs did not show any significant change in size after dispersion in cell culture medium, while a minimal increase in ζ-potential was observed. This suggests that protein adsorption, if present, leads to the formation of a thin or “soft” corona that does not substantially affect the hydrodynamic size. Consistently, no detectable changes in the PCL-NP size distribution were observed over time.

The complete DLS dataset, including all tested concentrations and incubation time points, is reported in Supplementary Table S3. Overall, the data indicate that NP suspensions were relatively stable under the tested conditions, supporting their suitability for comparative biological testing and providing a consistent basis for subsequent hazard identification across all experimental models.

### 3.2. Cytotoxicity in Monocultures of Caco-2 Cells

Cytotoxicity was first evaluated as an initial screening endpoint to identify non-lethal exposure conditions suitable for downstream functional and genotoxicity analyses. The results, shown in Figure S1A–C, indicated an absence of cytotoxic effects for all NP doses tested at both exposure times. Overall, cytotoxicity data supported the selection of exposure concentrations that maintained cell viability and were suitable for the investigation of subsequent tiers within the NPs hazard identification framework, including the maximum recommended concentration of 100 µg/mL for materials in nanoform.

### 3.3. Cell Internalization

**Figure 1:**
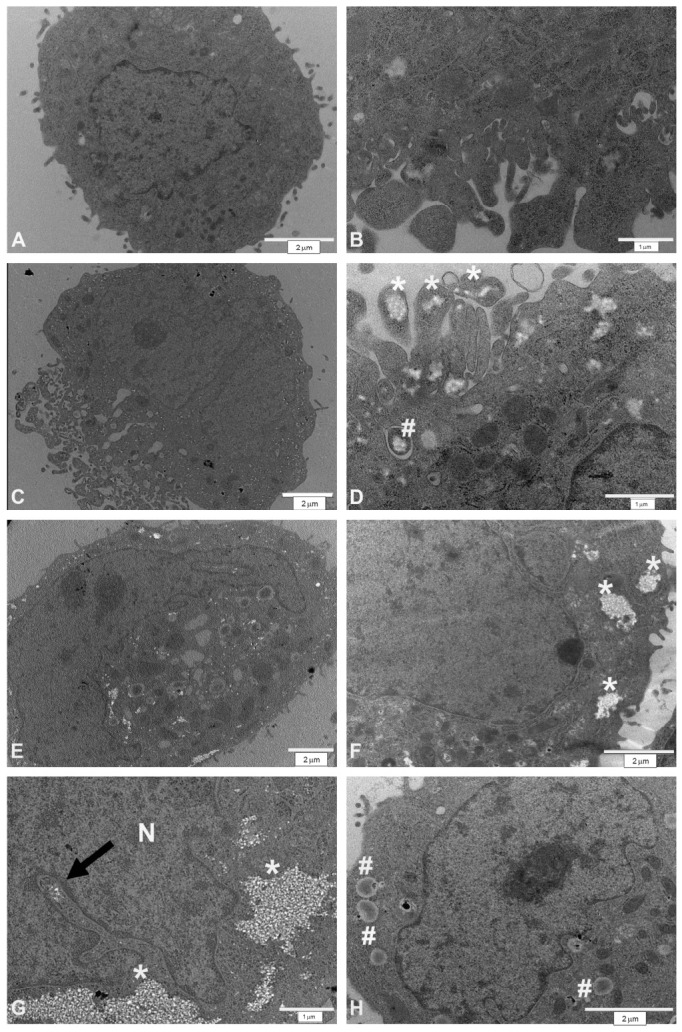
Ultrastructural analysis of interaction between CaCo-2 cells and PS-NPs 20 and 100 nm (100 μg/mL) for 1, 4, and 24 h. (**A**,**B**): untreated CaCo-2 cells; (**C**,**E**,**G**): CaCo-2 cells treated with PS-NPs 20 nm (100 μg/mL) for 1, 4, and 24 h, respectively. In (**G**), the arrow indicates small PS-NPs which are to be engulfed by the nucleus (N). Asterisk: agglomerated PS-NPs. (**D**,**F**,**H**): CaCo-2 cells treated with PS-NPs 100 nm (100 μg/mL) for 1, 4, and 24 h, respectively. Asterisk: agglomerated PS-NPs into microvilli; hashtag: agglomerated PS-NPs into lysosome.

**Figure 2:**
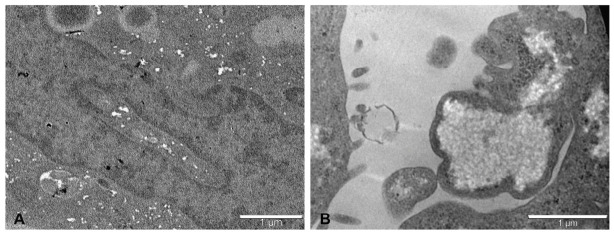
Details of TEM observations. (**A**) PS-NPs of 20 nm inside the nucleus of CaCo-2 cells. (**B**) cluster of PS-NPs into microvilli.

To further investigate NP–cell interactions at the subcellular level, ultrastructural analyses of Caco-2 monolayers were performed by transmission electron microscopy (TEM). Caco-2 control cells showed a well-preserved cytoplasm, a regular nucleus, and thick microvilli (Figure 1A,B). After treatment with 20 nm PS-NPs (100 μg/mL) for 1 h, CaCo-2 cells kept the cytoplasm unchanged. The PS-NPs entered through the microvilli and remained scattered and diffused within the cell without clumping (Figure 1C). The exposure up to 4 h showed many PS-NPs dispersed in the cytoplasm and in the perinuclear region (Figure 1E). After 24 h of interaction, many PS-NPs clumped together in large clusters in the cytoplasm (Figure 1G, asterisks), while the small ones moved closer to the nucleus to be incorporated (Figure 1G, arrow). The observations of cells treated with NPs with higher dimension (100 nm) for 1 h showed unmodified cytoplasm and agglomeration of PS-NPs inside the microvilli or lysosomal structure (Figure 1D, asterisks, and hashtag, respectively). After 4 h of interaction, the cells showed large clusters of NPs in the cytoplasm and near the nucleus (Figure 1F, asterisks), which tended to disappear after 24 h of interaction, leaving space for lysosomal degradation structures (Figure 1H, hashtag). None of the treatments used with 20 and 100 nm NPs at short or long times induced any changes or localizations in the mitochondria. Figure 2 shows two high magnification images of Caco-2 cells treated with 100 and 20 nm PS-NPs (Figure 2A) (Figure 2B) for 4 h. These observations suggested a nuclear-associated localization for the 20 nm PS-NPs (Figure 2A) and the entrapment of the 100 nm PS-NPs clusters within microvilli (Figure 2B). The TEM analysis provided predominantly qualitative rather than quantitative information regarding the intracellular localization and cellular internalization of NPs. The TEM measurements could not be performed for PCL-NPs, as this material is no longer commercially available from the manufacturer.

### 3.4. In Vitro Oxidative Stress

Oxidative stress in Caco-2 cells was assessed by quantifying the intracellular concentration of glutathione (GSH)—the major intracellular antioxidant—in its reduced and total forms, as well as the extracellular concentrations of GSH and related metabolites (CYS, CYSGLY, and HCY) involved in the cellular response to oxidative stress. Exposure of Caco-2 cells to 100 or 20 nm PS-NPs or 100 nm PCL-NPs at concentrations up to 100 µg/mL for up to 48 h did not significantly alter tGSHi, GSH redox status (rGSHi/tGSHi), or the levels of related thiols in the culture medium (Supplementary Table S2 and Figure S2). Regardless of the presence of nanoparticles, with increasing incubation time, tGSHi and rGSHi/tGSHi remained essentially stable, except for a slight decrease in tGSHi observed at 48 h (Supplementary Table S2). In contrast, tHCYe and tCYSGLYe increased while tCYSe and tGSHe tended to decrease (Supplementary Figure S2). These temporal trends indicate sustained cell metabolic activity over the 48 h experimental window. The absence of both a significant treatment effect and a significant interaction between time and treatment suggests that exposure to the NPs did not significantly modify the cell basal metabolism of glutathione and related thiols. Overall, these data suggest that, under the experimental conditions used, 100 nm PS-NPs, 20 nm PS-NPs, and 100 nm PCL-NPs do not induce detectable cellular oxidative stress in Caco-2 cells.

### 3.5. DNA Damage Evaluation by Comet Assay

Genotoxicity was evaluated as a key hazard endpoint to investigate whether NP exposure resulted in DNA damage in the absence of detectable cytotoxicity and alterations in cellular redox status. Cell viability, assessed at the time of cell harvesting using the Trypan Blue exclusion assay, resulted always greater than 85%. The genotoxic potential of the NPs was assessed using the alkaline comet assay to evaluate the primary DNA strand breaks and the FPG-modified comet assay to detect oxidized purines after 24 and 48 h of exposure, expressed as %Tail DNA.

**Figure 3:**
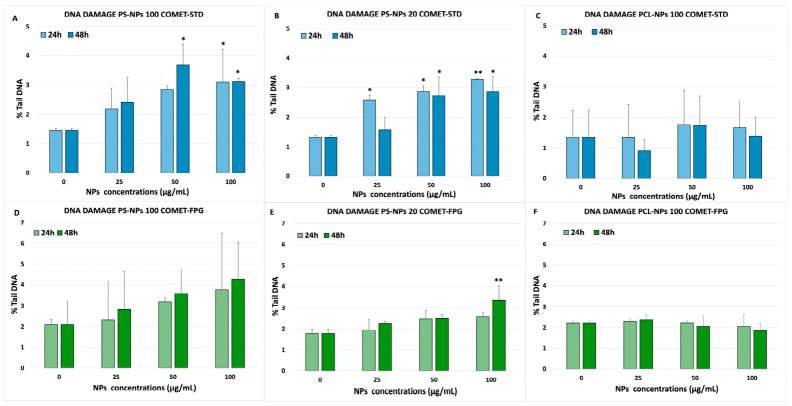
DNA damage detected by comet assay on Caco-2 cells after 24 and 48 h of treatment with 100 nm PS-NPs (**A**), 20 nm PS-NPs (**B**), and 100 nm PCL-NPs (**C**). Oxidative DNA damage detected with FPG after 24 and 48 h of treatment with 100 nm PS-NPs (**D**), 20 nm PS-NPs (**E**), and 100 nm PCL-NPs (**F**). Data are presented as mean ± standard deviation (SD). Statistical differences were determined using two-way ANOVA with Dunnett’s correction for multiple comparisons (* *p* <0.05; ** *p* <0.005) compared with the control group (0 µg/mL).

As shown in Figure 3A, 100 nm PS-NPs induced a significant increase in DNA damage at 100 μg/mL after 24 h of treatment and at the highest concentrations (50 and 100 μg/mL) after 48 h (*p* < 0.05; two-way ANOVA followed by Dunnett’s post hoc test). Compared with the control, the increase in %Tail DNA ranged from approximately 1.6 to 2.2 units (95% CI: 0.02–3.89) depending on concentration and exposure time.

Similarly, 20 nm PS-NPs induced a significant increase in %Tail DNA at all tested concentrations after 24 h (*p* < 0.05 at 25 and 50 μg/mL; *p* < 0.005 at 100 μg/mL). After 48 h of treatment, a DNA damage significant increase was observed at the highest concentrations (50 and 100 μg/mL) (*p* < 0.05; two-way ANOVA followed by Dunnett’s post hoc test) (Figure 3B). The observed increases in DNA damage ranged from approximately 1.3 to 2.0%Tail DNA units compared with controls (95% CI: 0.05–3.16).

The FPG-modified comet assay (Figure 3D,E) revealed a significant increase in oxidized purines exclusively for 20 nm PS-NPs after 48 h of treatment at the highest concentration tested (*p* < 0.005; two-way ANOVA followed by Dunnett’s post hoc test) (Figure 3E) with an increase in %Tail DNA, compared with the control, of approximately 1.6 units (95% CI: 0.49–2.68).

No DNA damage was detected at any dose or time point following exposure to 100 nm PCL-NPs, and the presence of FPG likewise did not reveal any increase in oxidized DNA bases (Figure 3C,F). The positive control, H_2_O_2_ (150 µM for 30 min), induced a significant increase in DNA damage and FPG-sensitive sites, consistent with the laboratory historical control range.

Differences in DNA damage levels were observed among the tested NPs, suggesting polymer- and size-dependent effects under the applied experimental conditions. These findings support the usefulness of genotoxicity related endpoints for investigating differential biological responses to NPs in intestinal models.

### 3.6. Barrier Integrity

Based on the absence of cytotoxicity at the selected doses, the effects of NPs on intestinal barrier integrity were assessed in vitro by measuring paracellular permeability using the LY assay. Only co-cultures exhibiting TEER values ≥ 150 Ω·cm^2^ were used for subsequent exposure to the tested NPs.

**Figure 4:**
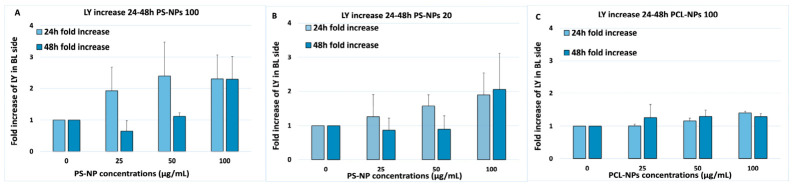
Lucifer yellow assay inserts Caco-2/HT-29/Raji-B 100 nm PS-NPs (**A**), 20 nm PS-NPs (**B**), and 100 nm PCL-NPs (**C**) at 24 h and 48 h compared with the control group (0 µg/mL). Data are presented as mean ± standard deviation (SD).

Exposure to 100 nm PS-NPs was associated with a slight increase in LY passage compared to the control at the highest concentrations after 24 h, while a comparable effect was observed only at the highest dose after 48 h (Figure 4A). Exposure to 20 nm PS-NPs showed a slight tendency toward an increase in LY passage after 24 h, with the increasing of the dose (Figure 4B). In both cases, LY permeability tended to decrease at 48 h at lowest doses, suggesting a partial recovery of barrier function.

No increase in paracellular permeability was observed after exposure to PCL-NPs, at both treatment times (Figure 4C).

Although none of the treatments resulted in statistically significant differences compared with the control, the observed trends may suggest a limited interaction of PS-NPs with the intestinal barrier and highlight the potential utility of functional permeability assays within a NAM-based testing strategy.

### 3.7. C. elegans Data

Building on the in vitro findings, the next tier of the NAM framework was designed to extend the hazard assessment into a whole-organism context, using the in vivo model *C. elegans* to capture organism-level responses and potential systemic effects, in line with the 3R principles.

In vivo experiments were performed to evaluate the effects of selected NPs on locomotion, which served as a standardized and easily quantifiable indicator of neuromuscular toxicity. To enhance reproducibility, locomotion was assessed in both solid and liquid media, measuring body bends per minute and thrashes per minute, respectively. In addition, activation of the antioxidant response was investigated. Synchronized L1 larvae were exposed to NPs at concentrations ranging from 0 to 100 µg/mL. Treatments were carried out on NGM agar plates at 20 °C for approximately three days, from the L1 larval stage to the young adult (YA) stage. For multigenerational experiments, exposure was maintained continuously across generations. Thus, while P0 animals were exposed from L1 to YA, F1 and F2 generations experienced prolonged cumulative exposure from the L1 stage of the parental P0 generation until reaching the YA stage of the respective generation.

No embryonic or larval lethality was observed for any of the tested NPs across all treatment conditions and exposure times.

#### 3.7.1. Effect of PS-NP (100 nm) Exposure

**Figure 5:**
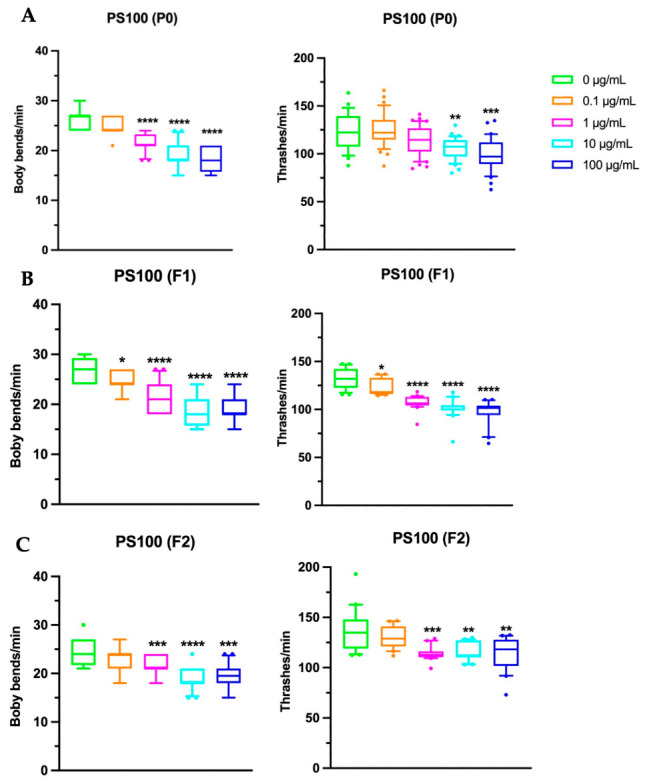
Effect of exposure to different concentrations of 100 nm PS-NPs on the locomotor behaviour of *C. elegans*, in the P0 (**A**), F1 (**B**), and F2 (**C**) generations. * *p* < 0.05; ** *p* < 0.005; *** *p* < 0.0005; **** *p* < 0.0001 (one-way ANOVA test with Dunnett’s correction for multiple comparisons) compared with non-treated nematodes (0 μg/mL). Boxes represent the 10th–90th percentile interval.

A clear dose-dependent reduction in both body bends and thrashes per minute was observed across all exposed generations (Figure 5). No major qualitative/quantitative differences were evident in the overall response pattern among generations. However, locomotor defects in the first treated (P0) generation became evident at 1 µg/mL in the body bend assay and only at the two highest concentrations in the thrashing assay (with a reduction of 31% in body bends and of 19% in head thrashes at the highest dose tested; *p* < 0.0001 and *p* < 0.0005 Dunnett’s post hoc test, respectively). The F1 generation showed a statistically significant reduction in both parameters already at the lowest concentration tested (with a reduction of 30% in body bends and of 25% in head thrashes, at the highest dose tested; *p* < 0.0001 Dunnett’s post hoc test, for both endpoints), suggesting increased sensitivity to PS-NPs upon two-generational exposure. Interestingly, F2 animals exhibited a weaker dose–response relationship in body bends compared with P0 and F1 worms, indicating a possible adaptive response or partial tolerance following prolonged multigenerational exposure across generations (with a reduction of 21% in body bends and of 16% in head thrashes, at the highest dose tested; *p* < 0.0005 and *p* < 0.005 Dunnett’s post hoc test, respectively) (Figure S3A).

**Figure 6:**
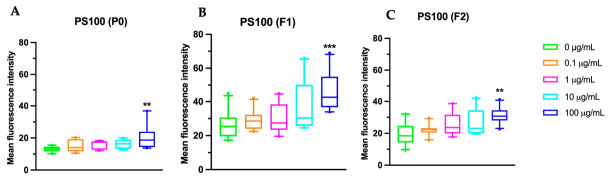

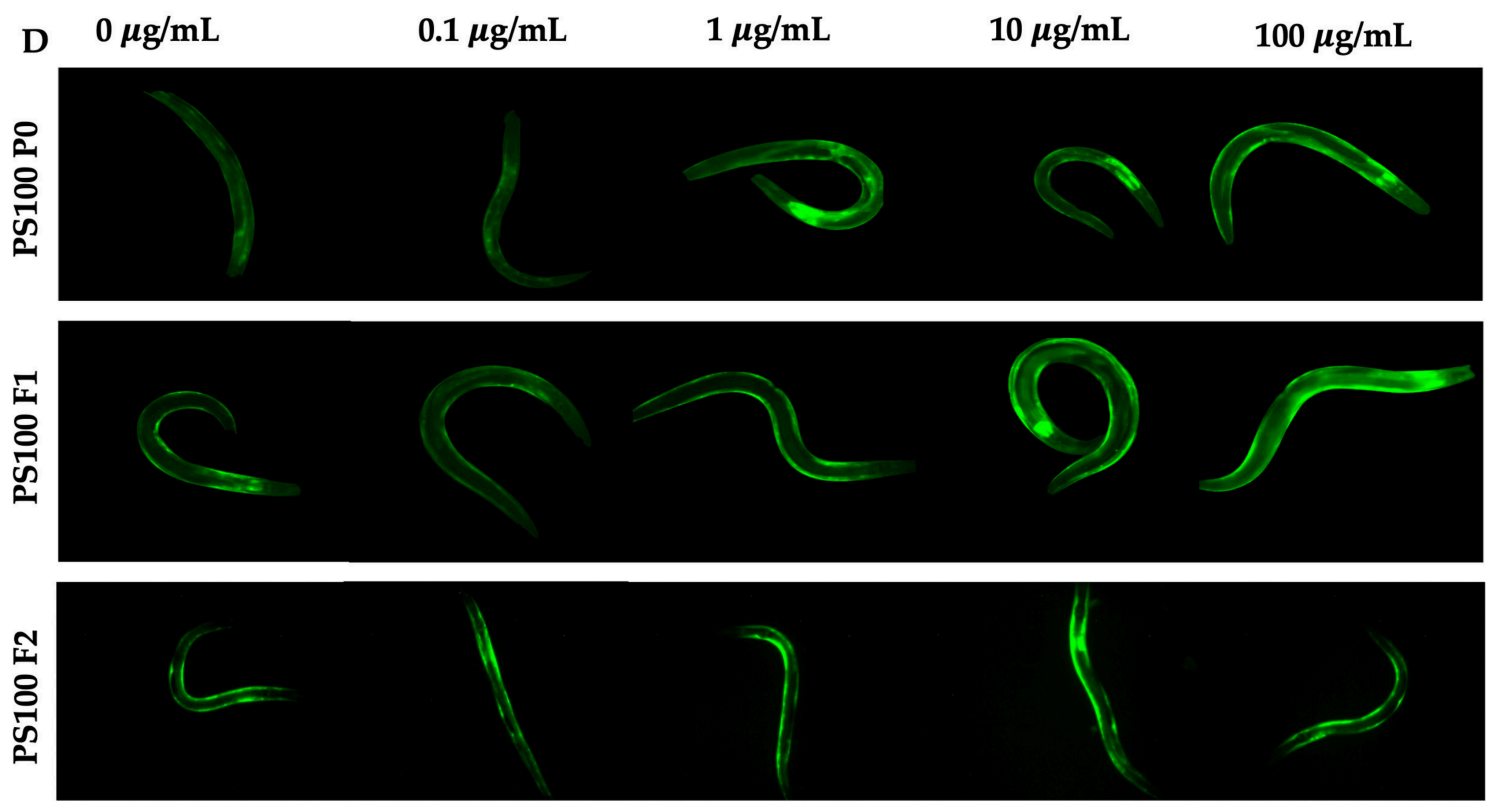
Mean fluorescence intensity in the *C. elegans* CL2166 strain following exposure to different concentrations of 100 nm PS-NPs in the P0 (**A**), F1 (**B**), and F2 (**C**) generations. ** *p* < 0.005; *** *p* < 0.0005 (one-way ANOVA test with Dunnett’s correction for multiple comparisons) compared with non-treated nematodes (0 μg/mL). Boxes represent the 10th–90th percentile interval. Representative fluorescence images are shown in (**D**). All images were acquired using identical microscope settings.

Oxidative stress was assessed using the transgenic strain CL2166, in which GFP expression is driven by the gst-4 promoter and reflects activation of the cellular oxidative stress response. A dose-dependent increase in fluorescence intensity was detected in all generations, although high inter-individual variability led to statistically significant differences only at the highest concentrations tested (with an increase in oxidative stress response of 58% in P0, 75% in F1 and of 58% in F2 at the highest dose tested; *p* < 0.005, *p* < 0.0005 and *p* < 0.005 Dunnett’s post hoc test, respectively) (Figure 6).

#### 3.7.2. Effect of PS-NPs (20 nm) Exposure

**Figure 7:**
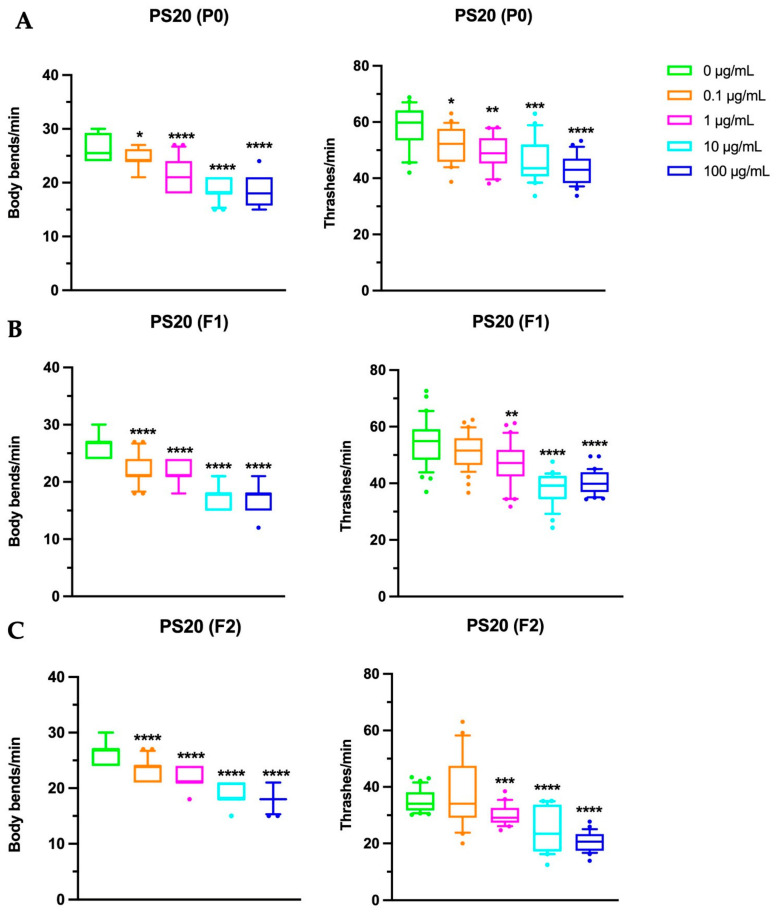
Effect of exposure to different concentrations of 20 nm PS-NPs on the locomotor behaviour of *C. elegans*, in the P0 (**A**), F1 (**B**), and F2 (**C**) generations. * *p* < 0.05; ** *p* < 0.005; *** *p* < 0.0005; **** *p* < 0.0001 (one-way ANOVA test with Dunnett’s correction for multiple comparisons) compared with non-treated nematodes (0 μg/mL). Boxes represent the 10th–90th percentile interval.

A clear dose-dependent reduction in both locomotor phenotypes was observed across all generations tested, and even the lowest concentration was sufficient to induce a significant impairment in the P0 generation (Figure 7) (with a reduction of 30% in body bends and of 26% in head thrashes, at the highest dose tested; *p* < 0.0001 Dunnett’s post hoc test, for both endpoints). Consistent with the pattern observed for 100 nm PS-NPs, exposure to 20 nm PS-NPs resulted in increased sensitivity in the F1 generation compared with P0 (with a reduction of 34% in body bends and of 26% in head thrashes, at the highest dose tested; *p* < 0.0001 Dunnett’s post hoc test, for both endpoints), while a partial recovery in body bends was observed in the F2 generation (Figure S3B) (with a reduction of 31% in body bends and of 42% in head thrashes, at the highest dose tested; *p* < 0.0001 and *p* < 0.0005 Dunnett’s post hoc test, respectively).

**Figure 8:**
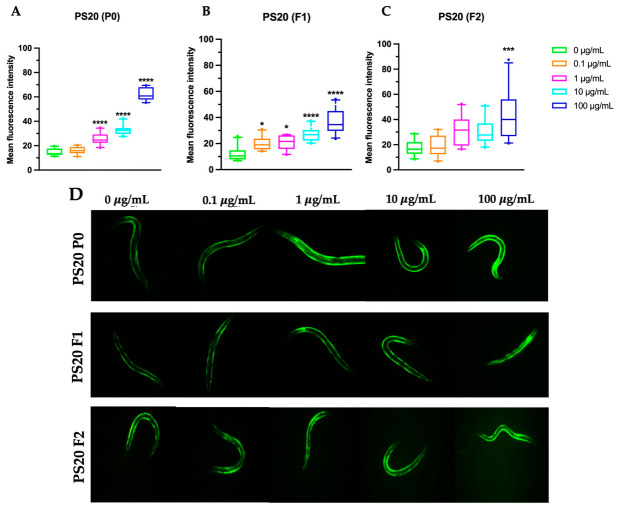
Mean fluorescence intensity in the *C. elegans* CL2166 strain following exposure to different concentrations of 20 nm PS-NPs in the P0 (**A**), F1 (**B**), and F2 (**C**) generations. * *p* < 0.05; *** *p* < 0.0005; **** *p* < 0.0001 (one-way ANOVA test with Dunnett’s correction for multiple comparisons) compared with non-treated nematodes (0 μg/mL) Boxes represent the 10th–90th percentile interval. Representative fluorescence images are shown in (**D**). All images were acquired using the same microscope settings.

Fluorescence analysis in the transgenic strain CL2166 revealed a strong, dose-dependent induction of the oxidative stress response, particularly in the initially exposed P0 generation (with a 300% increase in fluorescence intensity at the highest dose tested; *p* < 0.0001 Dunnett’s post hoc test) (Figure 8). 

**Figure 9:**
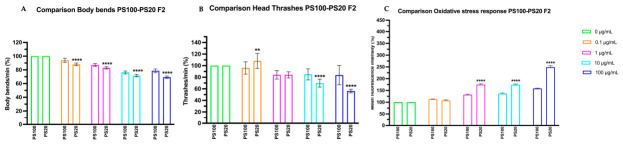
Comparison of the effects of exposure to different concentrations of 100 nm and 20 nm PS-NPs on body bends (**A**), head thrashes (**B**), and oxidative stress response (**C**) in *C. elegans* at the F2 generation. Data are presented as mean ± standard deviation (SD) ** *p* < 0.05; **** *p* < 0.0001 (Ordinary two-way ANOVA with Bonferroni’s multiple comparison tests, with a single pooled variance).

Moreover, the locomotor impairment induced by 20 nm PS-NPs was more pronounced than that observed with 100 nm PS-NPs, with a statistically significant greater reduction in locomotion detected in F2 at all doses, specifically an additional 11.5% reduction in body bends and a 35% reduction in head thrashes, at the highest dose tested (*p* < 0.0001 Bonferroni’s post hoc test) (Figure 9A,B). These findings support the notion that smaller PS-NPs exhibit higher toxicity, potentially due to their higher surface area-to-volume ratio and increased biological reactivity. In agreement with the locomotor data, exposure to 20 nm PS-NPs resulted in a significantly greater induction of the oxidative stress response compared with 100 nm PS-NPs across all examined generations, with an approximately 55% increase observed at the highest dose in the F2 generation (*p* < 0.0001 Bonferroni’s post hoc test) (Figure 9C).

#### 3.7.3. Effect of PCL-NPs (100 nm) Exposure

**Figure 10:**
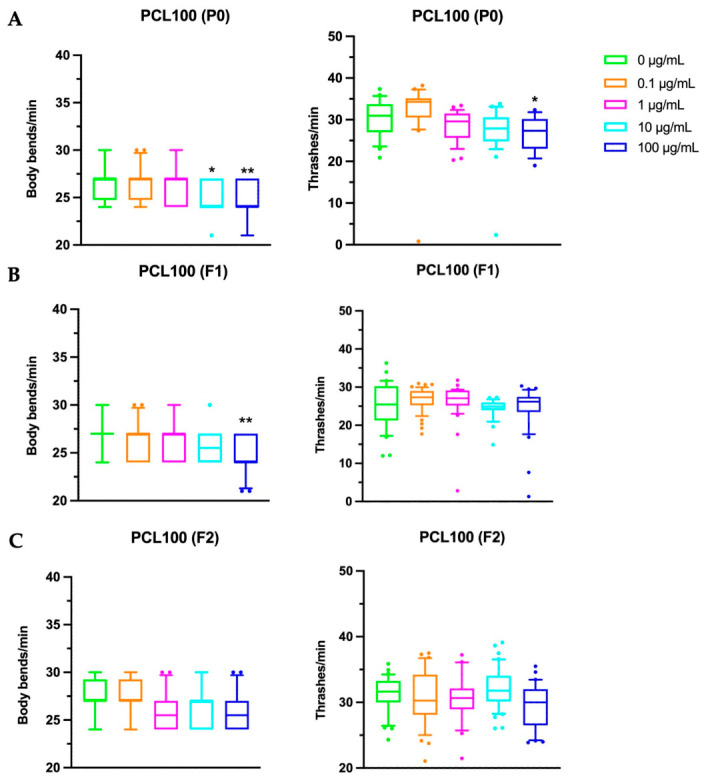
Effect of exposure to different concentrations of 100 nm PCL-NPs on the locomotor behaviour of *C. elegans*, in the P0 (**A**), F1 (**B**), and F2 (**C**) generations. * *p* < 0.05; ** *p* < 0.005 (one-way ANOVA test with Dunnett’s correction for multiple comparisons) compared with non-treated nematodes (0 μg/mL). Boxes represent the 10th–90th percentile interval.

Exposure to 100 nm PCL-NPs resulted in only mild effects on *C. elegans* locomotion compared with the impairment induced by PS-NPs of the same size (Figure 10). Locomotion assays across different concentrations showed no clear dose-dependent trend, with only a slight reduction in motility observed. A statistically significant decrease in body bends was detected only at the two highest concentrations in the P0 generation, while a significant reduction in head thrashes was observed exclusively at the highest concentration (with a reduction of 7.3% in body bends and of 12% in head thrashes (*p* < 0.005 and *p* < 0.05 Dunnett’s post hoc test, respectively) at the highest dose tested). In the F1 generation, a significant reduction was observed only for body bends at the highest concentration (with a deficit of 7.2%; *p* < 0.005 Dunnett’s post hoc test).

**Figure 11:**
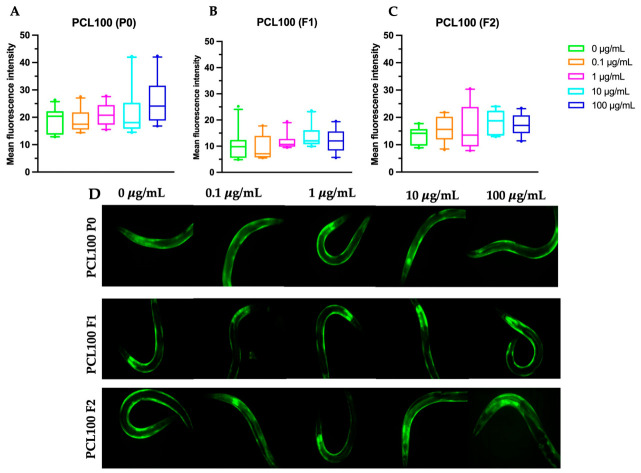
Mean fluorescence intensity in the *C. elegans* strain CL2166 following exposure to different concentrations of 100 nm PCL-NPs in the P0 (**A**), F1 (**B**), and F2 (**C**) generations compared with non-treated nematodes (0 μg/mL). Bands correspond to the 10th–90th percentile interval. Representative fluorescence images are shown in (**D**). All images were acquired using the same microscope settings.

Consistently, fluorescence analysis in the transgenic strain CL2166 revealed only a modest, non-significant tendency toward a dose-related increase in GFP intensity in the P0 generation, indicating limited activation of the oxidative stress response (Figure 11). In subsequent generations, this effect was further attenuated, with a near-complete recovery of fluorescence levels to those observed in control worms.

Additional statistical details for the *C. elegans* results, including mean differences, 95% confidence intervals (95% CI), and exact *p* values, are provided in the Supplementary Materials.

Overall, the in vivo responses to the biodegradable PCL-NPs were reduced compared with PS-NPs, in line with the comparative trends observed in the in vitro intestinal models. These findings support the biological relevance of the integrated NAM-based framework by demonstrating coherent responses across different experimental systems.

### 3.8. Results of the FAIRification

The preliminary FAIRification process began with an evaluation of the available templates on the Nanosafety data interface to ensure they could accommodate the key endpoint data studied in the project. The preliminary assessment indicates that the endpoints are divided into three different scenarios, depending on the availability of templates in the interface:

1. Available templates, to be used without modification: MTS, Comet assay, Barrier Integrity/Paracellular Permeability: (LY).
2. Templates requiring adaptation: particle size and ζ-potential measurements, oxidative stress assays.
3. Unavailable template, to be fully developed: *C. elegans* (Behavioural assays and oxidative stress response measurements).

## 4. Discussion

Studies investigating the biological effects of MNPs are still limited by the lack of standardized and validated approaches for elucidating their mechanisms of action, which represent a major barrier to accurately identify the potential health risks associated with MNPs [31,32]. In this study, a tiered testing strategy combining human intestinal in vitro models and the 3R-compliant organism *C. elegans* was applied to investigate NP-induced biological effects. This integrated approach enables the assessment of both cellular-level interactions and organism-level outcomes, thereby contributing to the development of more robust and harmonized frameworks for MNP hazard assessment. Overall, our findings underscore key cellular and physiological responses elicited by MNP exposure and highlight the critical role of particle size and material composition in determining biological responses.

PS-NPs were selected as reference materials due to their widespread use, well-characterized physicochemical properties, and extensive toxicological dataset [33,34,35,36,37], allowing direct comparison with previous studies and supporting method development. In parallel, biodegradable PCL-NPs were included to explore whether alternative materials exhibit distinct biological behaviours and potentially reduced hazard profiles. This comparative approach provides relevant insight into the influence of material composition on NP-induced biological effects.

The experimental design and exposure conditions were defined in accordance with current international recommendations for nanomaterial testing, ensuring appropriate particle characterization, dispersion quality, and reliable data interpretation [38,39]. The applied framework was selected to minimize assay interference and to support the generation of reproducible and comparable results across in vitro and in vivo models [40].

Ultrastructural analysis confirmed the cellular uptake of both 20 and 100 nm PS-NPs in Caco-2-based models, in agreement with previous reports [33,34,35,36,37]. Notably, TEM observations suggested that particle size influenced intracellular localization: while 100 nm PS-NPs accumulated mainly in the cytoplasm and in close proximity to the nucleus, 20 nm PS-NPs showed a nuclear-associated localization, particularly at longer exposure times. This size-dependent subcellular distribution suggest that smaller NPs possess an enhanced ability to access critical intracellular targets. Uptake studies for PCL-NPs could not be performed, as these NPs were no longer available; nevertheless, literature data have demonstrated their uptake in in vitro intestinal models [9].

Cytotoxicity, genotoxicity, and oxidative stress were evaluated using complementary assays in Caco-2 cells. None of the tested NPs induced significant cytotoxicity or oxidative stress, in line with previous findings [33,34,35,36,37,41,42]. Accordingly, exposure to NPs did not cause ultrastructural alterations in mitochondria, nor did the particles localize near these organelles. The observed primary DNA damage was overall moderate in magnitude. Notably, 20 nm PS-NPs induced significant effects starting from lower concentrations compared with 100 nm PS-NPs, suggesting a size-related difference in the biological response under the applied experimental conditions. Importantly, these effects were observed in the absence of cytotoxicity, supporting the interpretation of a genuine genotoxic response rather than a secondary consequence of cell toxicity. The lack of an increase in FPG-sensitive sites further indicates that the observed DNA damage was not primarily oxidative, corroborating the other observations reported in this study. This finding is in agreement with data by Rubio and colleagues, who likewise observed PS-NP-induced genotoxic effects only under non-FPG conditions [43]. Ultrastructural analyses further supported a size-dependent response, showing differences in intracellular localization, with 20 nm PS-NPs detected within the nucleus and 100 nm particles mainly confined to the cytoplasm. The earlier onset of the genotoxic response observed for 20 nm PS-NPs may be consistent with their greater intracellular and nuclear accessibility. Nevertheless, indirect mechanisms, including secondary cellular processes involved in genome maintenance, may also contribute to the observed DNA damage, as previously reported for other nanomaterials [44]. However, the presence of 100 nm PS-NPs in lysosomes and in close proximity to the nucleus could not exclude direct physical interaction between the NPs and nuclear DNA. Although evidence is currently lacking, a direct interaction between cytoplasmic NPs and DNA during mitosis, when the nuclear membrane is temporarily disassembled, cannot be ruled out [45]. Notably, PCL-NPs did not induce genotoxic effects, suggesting a possible influence of material composition on NP-induced biological responses. Despite the increasing scientific research focus on NPs, there is no consensus regarding the genotoxic effects of NPs, as findings across studies remain inconsistent, with some showing DNA damage induction [43,46] and others reporting no effects [34,35,47]. These discrepancies likely reflect differences in particle characteristics, exposure scenarios, and methodological approaches, underscoring the need for standardized testing strategies and harmonized protocols to enable more robust risk assessment.

The Caco-2/HT29-MTX/Raji-B tri-culture model was used to investigate NP-induced effects on intestinal barrier integrity [6,12]. The tendency toward an increase of paracellular permeability at 24 h exposure to both sizes of PS-NPs suggest a potential transient impairment of barrier integrity, partially reversed at 48 h, suggesting the activation of adaptive cellular responses, as previously proposed by Banaei and collaborators 2023 [48]. Previous studies have reported similar effects, suggesting that the observed increase in paracellular permeability could be related to alterations in tight junction (TJ) organization or dynamics [12]. TJs are highly dynamic structures that continuously adapt to physiological and pathological stimuli to preserve intestinal barrier integrity. Disruption of this adaptive capacity may represent a mechanism by which PS-NPs impair epithelial barrier function [49]. Although NP-mediated modulation of junctional proteins has been described in the literature, this mechanism was not specifically investigated in the present study, and no direct cause–effect relationship was established. In contrast, PCL particles induced a less pronounced increase in paracellular permeability compared with PS-NPs, suggesting a comparatively lower impact on epithelial barrier integrity. Overall, these findings suggest that material composition may represent a relevant factor in NP-induced intestinal barrier dysfunction. The overall in vitro results are in agreement with those of Boulée et al., who similarly evaluated the toxicity of PCL-NPs in in vitro intestinal models and reported no significant effects [9]. Although in vitro models provide valuable mechanistic insight into NP–cell interactions, they cannot fully capture organism-level effects. Within a tiered testing strategy, whole-organism models therefore represent a higher-level approach to evaluate cumulative and systemic responses to NP exposure. Accordingly, *C. elegans* was employed as a 3R-compliant in vivo model to assess NP-induced effects across generations, with a focus on locomotor behaviour and oxidative stress-associated responses.

In *C. elegans*, exposure to PS-NPs induced a clear dose-dependent reduction in locomotor activity, accompanied by activation of the oxidative stress response. These effects are consistent with previous studies reporting NP-induced neuromuscular and metabolic alterations in nematodes [26,50,51,52,53,54,55,56]. Although locomotor impairment and oxidative stress activation were observed concurrently, a direct causal relationship between these phenotypes cannot be assumed. Locomotor defects may arise from direct neurotoxicity or neuromuscular dysfunction, for instance through interference with synaptic signalling or body wall muscle function [54,56], while oxidative stress activation may represent a parallel systemic response to NP exposure [51,55]. Notably, 20 nm PS-NPs induced more pronounced effects than 100 nm PS-NPs, supporting a size-dependent toxicity profile at the organism level. Interestingly, multigenerational exposure did not lead to progressive sensitization (only a small effect was observed for 100 nm PS-NPs in F1); rather, F2 animals exhibited partial adaptation, suggesting that *C. elegans* may activate compensatory mechanisms upon prolonged exposure to PS-NPs.

The absence of oxidative damage in Caco-2 cells following NP exposure, together with the activation of the oxidative stress response observed in *C. elegans,* highlights fundamental differences between in vitro and in vivo models [57]. Intestinal epithelial systems possess robust antioxidant and detoxification mechanisms that may efficiently buffer low or transient oxidative insults, even in the presence of genotoxic effects. In contrast, *C. elegans*, as a whole-organism model, integrates systemic responses across multiple tissues and may therefore exhibit heightened sensitivity to sub-toxic stressors. In addition, the endpoints used in the two models capture distinct aspects of oxidative stress: biochemical measurements in Caco-2 cells reflect cell gluthathione metabolism, whereas the *gst-4*::GFP reporter in *C. elegans* represents a highly sensitive transcriptional readout of SKN-1/NRF2-dependent stress response activation, which may be triggered even in the absence of overt oxidative damage. Furthermore, organism-level exposure may result in cumulative uptake and tissue-specific accumulation of NPs, leading to localized or transient oxidative challenges that are not detectable in simplified in vitro systems. Differences in exposure dynamics and duration between the in vitro and in vivo models (48 h vs. multigenerational exposure, respectively) may also contribute to the observed divergence in oxidative stress-associated responses. Moreover, in *C. elegans*, NPs are exposed to digestive enzymes, microbes, and variable pH in the gut, which can alter their surface chemistry and increase their biological reactivity compared to in vitro conditions. Indirect ROS generation may also arise from organism-level perturbations such as altered nutrient uptake or mechanical stress, which trigger systemic stress responses and secondary increases in endogenous ROS production rather than direct pro-oxidant activity of the material.

Finally, exposure to biodegradable PCL-NPs resulted in milder biological effects compared with PS-NPs, consistently across in vitro and in vivo models. In *C. elegans*, PCL-NPs induced only a slight reduction in locomotor activity and a weak, non-significant activation of the oxidative stress response, in agreement with the absence of cytotoxic, genotoxic, and barrier-disrupting effects observed in vitro. Despite the lack of literature data on *C. elegans* exposure to PCL, several studies have reported that biodegradable plastics can induce toxic effects [58,59]. However, given the limited availability of data on biodegradable NPs, further studies are required before definitive conclusions regarding their safety can be drawn.

Implementing FAIR data principles is essential for ensuring the evaluation and reuse of scientific results. The Template Wizard was developed to assist researchers in creating standardized, machine-readable templates for experimental data and metadata, simplifying the adoption of FAIR practices. Originally designed from over a decade of EU-funded nanosafety research, these templates are versatile and have been adapted for diverse fields such as microplastics, nanoplastics, and advanced materials, covering more than 60 types of assays [16,30].

As a case study within the EU Gov4Nano project, the FAIRification of in vitro Comet assay data demonstrated how the eNanoMapper model (Nanosafety data interface, webform) and the Minimum Information for Reporting Comet Assay (MIRCA) reporting standards [60] can enhance data quality, integration, comparability, and reusability for predictive modelling applications [61]. Similarly, at this stage of the present project where data has just been produced and the FAIRification process is underway, various scenarios relating to the availability of templates in eNanoMapper across key endpoints have been identified (i.e., available, available with adaptation, or unavailable templates).

FAIRification of the experimental results from this project will allow future analysis on the critical nanoplastics characteristics which may impact experimental outcomes. This understanding will contribute to the standardization of methods and protocols for NP toxicity assessment. FAIRification of the experimental results will facilitate future analyses of NP characteristics influencing experimental outcomes and contribute to the standardization of methods for NP toxicity assessment. Increased data accessibility and quality may also improve the understanding and reusability of these results in future studies.

Some limitations of the present study should be acknowledged. First, delivered dose quantification and endotoxin testing of the nanomaterials were not performed. Nevertheless, dynamic light scattering and stability analyses showed no significant nanoparticle aggregation under the experimental conditions, suggesting that given the small hydrodynamic size of the nanoparticles, only minor deviations between nominal and delivered dose due to sedimentation are expected. In addition, the absence of overt cytotoxicity, the high cell viability, the lack of marked oxidative stress responses even at the highest concentration and longest exposure time, and the absence of morphological signs of cellular stress observed by TEM collectively indicate that substantial endotoxin-related interference is unlikely under the applied conditions [62]. Future studies should nonetheless include dedicated assessments of delivered dose and endotoxin contamination. Second, the limited commercial availability of PCL-NPs prevented the performance of TEM internalization studies, thereby precluding a detailed ultrastructural evaluation of particle–cell interactions and subcellular localization. The TEM observations carried out in this study provided mainly qualitative information on ultrastructural cell alterations and NP localization, without quantitative assessment of particle uptake or intracellular distribution. In addition, the limited number of independent replicates may have reduced the statistical power of the study. Furthermore, the overall adopted exposure scenario may not fully reflect environmentally realistic exposure conditions. Moreover, the biodegradation kinetics, environmental persistence, and potential bioaccumulation of NPs were not directly investigated in the present study; therefore, the observed milder effects of PCL-NPs, compared with PS-NPs, should be interpreted only within the specific experimental conditions and endpoints evaluated. The absence of inflammatory markers represents a limitation of the present study and restricts the interpretation of the biological responses, particularly in relation to potential immune-related mechanisms. Although inflammatory and immunotoxicological endpoints were beyond the experimental scope of this work, increasing evidence indicates that immune and inflammatory pathways may represent important determinants of nanoplastic toxicity in vivo and should therefore be considered in future NAM-based testing strategies [11]. Finally, the FAIRification process applied in this study should be considered preliminary, and further efforts will be required to ensure full compliance with FAIR data principles. Overall, these limitations highlight the need for further studies integrating advanced particle characterization, quantitative uptake analyses, inflammatory endpoints, and environmentally relevant exposure scenarios to strengthen the application of NAM-based approaches for nanoplastic hazard assessment.

## 5. Conclusions

In summary, several research gaps and methodological challenges remain in the assessment of MNP toxicity, particularly with respect to the generation of data suitable for regulatory decision-making. A comprehensive and fit-for-purpose human health risk assessment framework for MNPs is still lacking, and the currently available toxicological evidence remains limited, especially regarding the long-term effects of persistent particles. Further targeted research is therefore needed to clarify toxicity mechanisms and to generate reliable, regulatory relevant data. Within this context, the tiered NAM-based approach described here may contribute to improving the consistency and interpretability of future studies. The consideration of FAIR-oriented data practices may further support data transparency and reusability, facilitating the integration of emerging evidence and understanding the key phenomena. Overall, such approaches may help inform future risk assessment and risk mitigation strategies, including the evaluation and prioritization of alternative materials within a safer-by-design perspective.

## Figures and Tables

**Table 1:** NP properties.

Property	PS-NP100	PS-NP20	PCL-NP100
Material	Polystyrene	Polystyrene	Polycaprolactone
**Surface chemistry**	unmodified	unmodified	unmodified
**Nominal diameter**	100 nm	20 nm	100 nm
**Density (material)**	1.05 g/cm^3^	1.05 g/cm^3^	1.146 g/cm^3^
**Refractive Index**	1.59	1.59	1.46
**Stock dispersant**	Water	Water	Water
**Stock concentration (mass)**	100 mg/mL	10 mg/mL	10 mg/mL
**Stock concentration (number) ^a^**	1.828 × 10^14^/mL	2.275 × 10^15^/mL	1.669 × 10^13^/mL

^a^ = Estimated from material density, mass concentration and diameter.

**Table 2:** NP characterization by Dynamic Light Scattering. Measurements were performed on freshly prepared dispersions in water or cell culture medium (DMEM + 10% FBS), as well as on cell culture medium dispersions incubated under the same conditions and for the same times used in the in vitro experiments.

Tested NPs	Incubation Time	Conc μg/mL	Dispersant	Hydrodynamic Size (nm)	PDI	ζ-Potential (mV)
PS-NP100	t = 0 h	100	H_2_O/1 mM NaCl *	103.5 ± 0.3 ^a^	0.02 ± 0.018	−45.5 ± 0.9
	t = 0 h	100	DMEM + 10% FBS	126.7 ± 1.3 ^a^	0.12 ± 0.023	−9.5 ± 1.6
	t = 48 h	100	DMEM + 10% FBS	152.8 ± 1.7 ^a^	0.22 ± 0.009	
PS-NP20	t = 0 h	100	H_2_O/1 mM NaCl *	23.3 ± 0.1 ^a^	0.10 ± 0.015	−44.6 ± 3.4
	t = 0 h	100	DMEM + 10% FBS	34.3 ± 0.1 ^a^	0.30 ± 0.003	−11.3 ± 0.9
	t = 48 h	100	DMEM + 10% FBS	30.4 ± 0.3 ^a^	0.29 ± 0.003	
PCL-NP100	t = 0 h	100	H_2_O/1 mM NaCl *	116.1 ± 0.1 ^a^ 130.7 ± 3.3 ^b^	0.131 ± 0.016	−2.80 ± 0.15
	t = 0 h	100	DMEM + 10% FBS	131.6 ± 3.9 ^b^	0.508 ± 0.007	−3.49 ± 0.06
	t = 48 h	100	DMEM + 10% FBS	136.8 ± 1.5 ^b^	0.494 ± 0.005	

^a^ = Z-average; ^b^ = Peak 1 size from the intensity-weighted size distributions. * = 1 mM NaCl used instead of water for ζ-potential measurements.

## Data Availability

The original contributions presented in this study are included in the article/Supplementary Materials. Further inquiries can be directed to the corresponding author.

## Supplementary Materials

The following supporting information can be downloaded at: https://www.mdpi.com/article/10.3390/nano16110668/s1, Figure S1: Cytotoxicity in monocultures of Caco-2 cells; Figure S2: Oxidative stress; Figure S3: Comparison of Body bends alterations in P0-F2 after exposure to PS-NPs; Figure S4: Comparison of Oxidative stress response in P0-F2 after exposure to PS-NPs; Table S1: Nanoplastic Particle Complete Characterization; Table S2: In vitro Oxidative stress; Table S3: Number of body bends per minute; Table S4: Number of head thrashes per minute; Table S5: Oxidative stress response in CL2166 nematodes.

## Abbreviations

The following abbreviations are used in this manuscript:

CYS	Cysteine
CYSGLY	Cysteinylglycine
DLS	Dynamic Light Scattering
FAIR	Findable, accessible, interoperable, and reusable
FPG	Formamidopyrimidine [fapy]-DNA glycosylase
GFP	Green Fluorescent Protein
GSH	Glutathione
HCY	Homocysteine
LY	Lucifer yellow
MNPs	Micro- and nanoplastics
MPs	Microplastics
NAMs	New Approach Methodologies
NGM	Nematode Growth Medium
NPs	Nanoplastics
PCL	Polycaprolactone nanoplastics
PS-NPs	Polystyrene nanoplastics
ROS	Reactive Oxygen Species
SOP	Standard Operative Procedure
TEER	Trans-epithelial Electrical Resistance
