# Supplementary material for: Developing a New Approach Methodology Framework to Assess Biological Responses to Nanoplastics: Insights from Polystyrene and Biodegradable Particles
Source: Nanomaterials (Basel). 2026 May 26;16(11):668. doi: 10.3390/nano16110668 (PMC13258071; doi:10.3390/nano16110668)
Supplement: Supplementary file 1 [file nanomaterials-16-00668-s001.zip › nanomaterials-4303429-supplementary.pdf]

Table S1. Nanoplastic Particles Complete Characterization

Tested NPs	Incubation Time	Conc µg/ml	Dispersant	Hydrodynamic size (nm)	PDI	ζ-potential (mV)
**PS-NP100**	t=0h	100	H2O / 1 mM NaCl*	103.5 ± 0.3 [a]	0.02± 0.018	-45.5 ± 0.9
		100	DMEM + 10% FBS	126.7 ± 1.3 [a]	0.12± 0.023	-9.5 ± 1.6
	t=4h	25	DMEM + 10% FBS	107.3 ± 0.7[a]	0.24± 0.002	
		50	DMEM + 10% FBS	117.0 ± 0.8[a]	0.16± 0.004	
		100	DMEM + 10% FBS	124.7 ± 0.5[a]	0.11± 0.018	
	t=24h	25	DMEM + 10% FBS	118.6 ± 0.9[a]	0.27± 0.004	
		50	DMEM + 10% FBS	131.9 ± 1.6[a]	0.20± 0.008	
		100	DMEM + 10% FBS	141.0 ± 0.5[a]	0.16± 0.003	
	t=48h	25	DMEM + 10% FBS	134.9 ± 1.4[a]	0.28± 0.007	
		50	DMEM + 10% FBS	145.7 ± 1.4[a]	0.25± 0.011	
		100	DMEM + 10% FBS	152.8 ± 1.7[a]	0.22± 0.009	
**PS-NP20**	t=0h	100	H2O / 1 mM NaCl*	23.3 ± 0.1[a]	0.10± 0.015	-44.6 ± 3.4
		100	DMEM + 10% FBS	34.3 ± 0.1[a]	0.30± 0.003	-11.3 ± 0.9
	t=4h	25	DMEM + 10% FBS	18.9 ± 0.1[a]	0.42± 0.003	
		50	DMEM + 10% FBS	22.1 ± 0.4[a]	0.36± 0.038	
		100	DMEM + 10% FBS	28.0 ± 0.3[a]	0.28± 0.003	
	t=24h	25	DMEM + 10% FBS	19.6 ± 0.1[a]	0.39± 0.003	
		50	DMEM + 10% FBS	22.5 ± 0.1[a]	0.39± 0.002	

		100	DMEM + 10% FBS	28.4 ± 0.2 [a]	0.28± 0.007	
	t=48h	25	DMEM + 10% FBS	20.9 ± 0.1 [a]	0.42± 0.002	
		50	DMEM + 10% FBS	25.0 ± 0.1 [a]	0.38± 0.004	
		100	DMEM + 10% FBS	30.4 ± 0.3 [a]	0.29± 0.003	

**PCL-NP100**	t=0h	100	H2O / 1 mM NaCl*	116.1 ± 0.1 [a] 130,7 ± 3,3 [b]	0.131 ± 0.016	-2,80 ± 0,15
		100	DMEM + 10% FBS	131,6 ± 3,9 [b]	0.508± 0.007	-3,49 ± 0,06
	t=24h	25	DMEM + 10% FBS	124.0 ± 6.3 [b]	0.636 ± 0.005	
		50	DMEM + 10% FBS	132.7 ± 5.7 [b]	0.598 ± 0.050	
		100	DMEM + 10% FBS	137.8 ± 1.1 [b]	0.486 ± 0.005	
	t=48h	25	DMEM + 10% FBS	133.3 ± 7.7 [b]	0.639 ± 0.033	
		50	DMEM + 10% FBS	133.5 ± 6.4 [b]	0.570 ± 0.003	
		100	DMEM + 10% FBS	136.8 ± 1.5 [b]	0.494 ± 0.005	

Cytotoxicity in monocultures of Caco-2 cells

The MTS assay measures cellular metabolic activity as an indicator of cell viability and cytotoxicity, based on the mitochondrial enzymatic reduction of the MTS tetrazolium compound to a soluble formazan product in metabolically active cells. The effects on cell viability following exposure to NPs were assessed in Caco-2 monocultures, in accordance with the NanoValid SOP.

The results, shown in Figure S1A, S1B, and S1C, indicated an absence of cytotoxic effects for all tested NPs at both exposure times (ANOVA, with Dunnett' test).

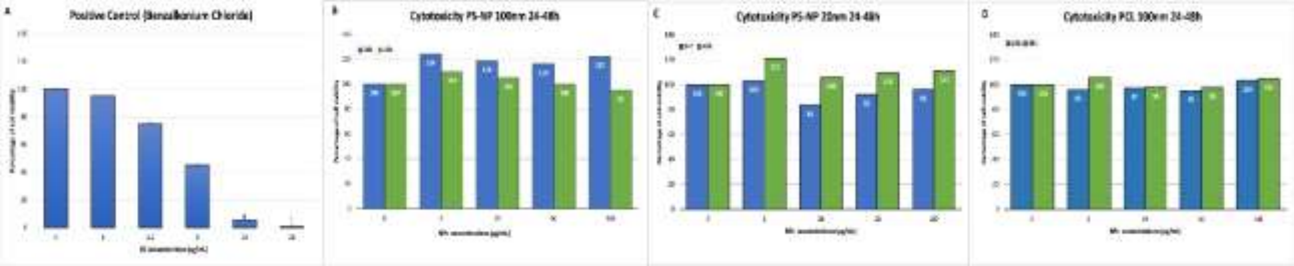

Figure S1: MTS cytotoxicity assay after Caco-2 mono-culture cells treatment with 100 nm PS-NPs (A), 20 nm PS-NPs (B) and 100 nm PCL-NPs (C). Benzalkonium Chloride was used as a positive control. Doses and times of treatment were selected according to NanoValid protocol. Data are presented as mean ± standard deviation (SD)

Table S2. In vitro Oxidative stress. Cell total glutathione (tGSHi) and reduced to total glutathione ratio (rGSHi/tGSHi) after Caco-2 cell exposure to nanoplastics.

Treatment	NP dose (µg/ml)	$tGSH_i$ (nmol/mg protein)			$rGSH_i/tGSH_i$ (%)		
		4h	24h	48h	4h	24h	48h
**PS-NP100**	0	40.2 ± 5.3	38.3 ± 1.7	33.5 ± 3.6	94 ± 5 %	87 ± 4 %	98 ± 11 %
	25	39.3 ± 4.1	37.6 ± 1.5	34.1 ± 0.4	90 ± 10 %	92 ± 10 %	103 ± 5 %
	50	39.2 ± 2	39.5 ± 0.3	32.9 ± 3.4	95 ± 6 %	94 ± 5 %	102 ± 10 %
	100	38.8 ± 3.5	36.8 ± 3.6	33.4 ± 2.8	93 ± 2 %	102 ± 8 %	97 ± 11 %
**$KBrO_3$[a]**		8.8 ± 2***			92 ± 13 %		
**PS-NP20**	0	35.8 ± 1.3	37.6 ± 3.2	30.5 ± 4.8	94 ± 2 %	93 ± 2 %	95 ± 4 %
	25	36.6 ± 2.3	35.8 ± 1.1	35.7 ± 0.5	96 ± 2 %	100 ± 8 %	98 ± 1 %
	50	34.4 ± 2.4	37 ± 1.9	34.2 ± 4.6	94 ± 8 %	100 ± 8 %	102 ± 5 %
	100	36.5 ± 0.6	36.4 ± 1.5	33.3 ± 0.6	96 ± 6 %	96 ± 2 %	95 ± 2 %
**$KBrO_3$[a]**		14.5 ± 2.2***			97 ± 8 %		
**PCL-NP100**	0	41.4 ± 2.9	40.3 ± 1.5	42.3 ± 1.1	104 ± 0 %	101 ± 6 %	103 ± 18 %
	25	40.6 ± 1.6	41.5 ± 3.5	40.3 ± 4.6	96 ± 5 %	100 ± 3 %	104 ± 5 %
	50	42.2 ± 1.1	40.1 ± 0.3	43.2 ± 2.3	104 ± 0 %	98 ± 2 %	104 ± 0 %
	100	39.7 ± 0.5	42.2 ± 1.3	41.2 ± 5.3	102 ± 0 %	94 ± 3 %	102 ± 0 %
**$KBrO_3$[a]**		17.5 ± 6.6**			97 ± 4 %		

Data are presented as mean ± SD. [a] Positive control: 40 mM KBrO3 in PBS for 30 minutes. $tGSH_i$ showed an overall slight decrease at 48h (main effect of time: $p < 0.05$; specifically $p < 0.01$ and $p = 0.059$ within PS-NP100 and PS-NP20 groups, respectively). No significant effects of NP treatment or time x treatment interaction were observed for either $tGSH_i$ or $rGSH_i/tGSH_i$ ratio (two-way ANOVA). **$p < 0.01$ and *** $p < 0.001$ vs. untreated control at 4h (one-way ANOVA with Dunnett's post-hoc test).

Table S3. Number of body bends per minute in N2 animals exposed or not to the selected NPs across multiple generations.

NPs	Dose (µg/mL)	Generation	Body bends/min	*p*-value	N
**PS-NPs 100 nm**					
	-	P0	26.4		20
	0.1	P0	24.7	0.0943	20
	1	P0	21.4	<0.0001	20
	10	P0	19.0	<0.0001	20
	100	P0	18.1	<0.0001	20
	-	F1	26.7		20

	0.1	F1	24.6	0.0365	20
	1	F1	21.4	<0.0001	20
	10	F1	18.6	<0.0001	20
	100	F1	18.7	<0.0001	20
	-	F2	24.6		20
	0.1	F2	23.1	0.2694	20
	1	F2	21.4	0.0006	20
	10	F2	18.7	<0.0001	20
	100	F2	19.3	0.0002	20
**PS-NPs 20 nm**					
	-	P0	25.3		20
	0.1	P0	24.3	0.0499	20
	1	P0	21.7	<0.0001	20
	10	P0	19.0	<0.0001	20
	100	P0	18.6	<0.0001	20
	-	F1	26.2		20
	0.1	F1	22.3	<0.0001	20
	1	F1	21.4	<0.0001	20
	10	F1	17.7	<0.0001	20
	100	F1	17.2	<0.0001	20
	-	F2	26.4		20
	0.1	F2	23.2	<0.0001	20
	1	F2	21.9	<0.0001	20
	10	F2	18.9	<0.0001	20
	100	F2	18.3	<0.0001	20
**PCL-NPs 100 nm**					
	-	P0	26.7		20
	0.1	P0	26.5	0.9900	20
	1	P0	26.1	0.7300	20
	10	P0	24.9	0.0163	20
	100	P0	24.7	0.0080	20

	-	F1	27.0		20
	0.1	F1	26.1	0.3900	20
	1	F1	26.2	0.5514	20
	10	F1	25.6	0.0958	20
	100	F1	25.0	0.0710	20
	-	F2	27.1		20
	0.1	F2	27.2	1	20
	1	F2	25.8	0.1347	20
	10	F2	26.0	0.3195	20
	100	F2	26.0	0.1347	20

NPs, nanoplalstics; PS-NPs, polystyrene nanoplalstics; PCL-NPs, polycaprolactone nanoplastics; N=number of animals tested. Statistical analysis was performed using one-way ANOVA test with Bonferroni correction for multiple comparisons

Table S4. Number of head thrashes per minute in N2 animals exposed or not to the selected NPs across multiple generations.

NPs	Dose (µg/mL)	Generation	Thrashes/min	*p*-value	N
**PS-NPs 100 nm**					
	-	P0	127.56	-	20
	0.1	P0	122.6	0.97	20
	1	P0	113.83	0.08	20
	10	P0	106.72	0.0015	20
	100	P0	100.50	0.0006	20
	-	F1	137.30	-	20
	0.1	F1	130.6	0.0126	20
	1	F1	114.5	<0.0001	20
	10	F1	115.7	<0.0001	20
	100	F1	113.87	<0.0001	20
	-	F2	136.12	-	20
	0.1	F2	132.67	0.5815	20
	1	F2	115.6	0.0005	20

	10	F2	116.8	0.0025	20
	100	F2	114.3	0.0031	20
**PS-NPs 20 nm**					
	-	P0	58.24	-	20
	0.1	P0	52.19	0.0189	20
	1	P0	49.38	0.0026	20
	10	P0	46.39	0.0006	20
	100	P0	44.48	<0.0001	20
	-	F1	54.5	-	20
	0.1	F1	51.27	0.1700	20
	1	F1	46.7	0.0013	20
	10	F1	37.7	<0.0001	20
	100	F1	40.34	<0.0001	20
	-	F2	34.4	-	20
	0.1	F2	37.2	0.7592	20
	1	F2	28.97	0.0002	20
	10	F2	24.01	<0.0001	20
	100	F2	19.33	<0.0001	20
**PCL-NPs 100 nm**					
	-	P0	30.26	-	20
	0.1	P0	31.95	0.8565	20
	1	P0	28.49	0.4164	20
	10	P0	27.4	0.2174	20
	100	P0	26.59	0.0172	20

	-	F1	26.39	-	20
	0.1	F1	26.77	0.3951	20
	1	F1	26.35	0.8570	20
	10	F1	25.28	0.8979	20
	100	F1	24.42	0.9423	20
	-	F2	31.18	-	20
	0.1	F2	30.74	0.9485	20
	1	F2	31.79	0.8459	20
	10	F2	32.17	0.3233	20
	100	F2	30.10	0.0728	20

NPs, nanoplalstics; PS-NPs, polystyrene nanoplalstics; PCL-NPs, polycaprolactone nanoplastics; N=number of animals tested. Statistical analysis was performed using one-way ANOVA test with Bonferroni correction for multiple comparisons

Table S5. Oxidative stress response in CL2166 animals exposed or not to the selected NPs across multiple generations.

NPs	Dose (µg/mL)	Generation	Mean fluorescence intensity	*p*-value	N
**PS-NPs 100 nm**					
	-	P0	13.07	-	10
	0.1	P0	15.20	0.7141	10
	1	P0	15.66	0.5647	10
	10	P0	16.20	0.3985	10
	100	P0	20.66	0.0034	10
	-	F1	26.58	-	10

	0.1	F1	29.08	0.9550	10
	1	F1	30.60	0.8058	10
	10	F1	37.22	0.0906	10
	100	F1	46.40	0.0004	10
	-	F2	19.71	-	10
	0.1	F2	22.13	0.8264	10
	1	F2	25.99	0.1210	10
	10	F2	26.86	0.0635	10
	100	F2	31.22	0.0012	10
**PS-NPs 20 nm**					
	-	P0	14.83	-	10
	0.1	P0	16.21	0.8712	10
	1	P0	25.63	<0.0001	10
	10	P0	32.75	<0.0001	10
	100	P0	62.14	<0.0001	10
	-	F1	12.03	-	10
	0.1	F1	20.08	0.0244	10
	1	F1	20.66	0.0144	10
	10	F1	27.04	<0.0001	10
	100	F1	36.78	<0.0001	10
	-	F2	17.65	-	10
	0.1	F2	18.90	0.9983	10
	1	F2	30.81	0.0814	10
	10	F2	30.73	0.0840	10

	100	F2	43.89	0.0001	10
**PCL-NPs 100 nm**					
	-	P0	18.87	-	10
	0.1	P0	18.65	>0.9999	10
	1	P0	20.97	0.8927	10
	10	P0	22.05	0.6724	10
	100	P0	24.60	0.0739	10
	-	F1	10.50	-	10
	0.1	F1	9.54	0.9721	10
	1	F1	11.82	0.9166	10
	10	F1	13.85	0.3100	10
	100	F1	12.01	0.8740	10
	-	F2	13.26	-	10
	0.1	F2	15.57	0.7079	10
	1	F2	16.47	0.4413	10
	10	F2	18.55	0.0843	10
	100	F2	17.22	0.2632	10

NPs, nanoplalstics; PS-NPs, polystyrene nanoplalstics; PCL-NPs, polycaprolactone nanoplastics; N=number of animals tested. Statistical analysis was performed using one-way ANOVA test with Bonferroni correction for multiple comparisons

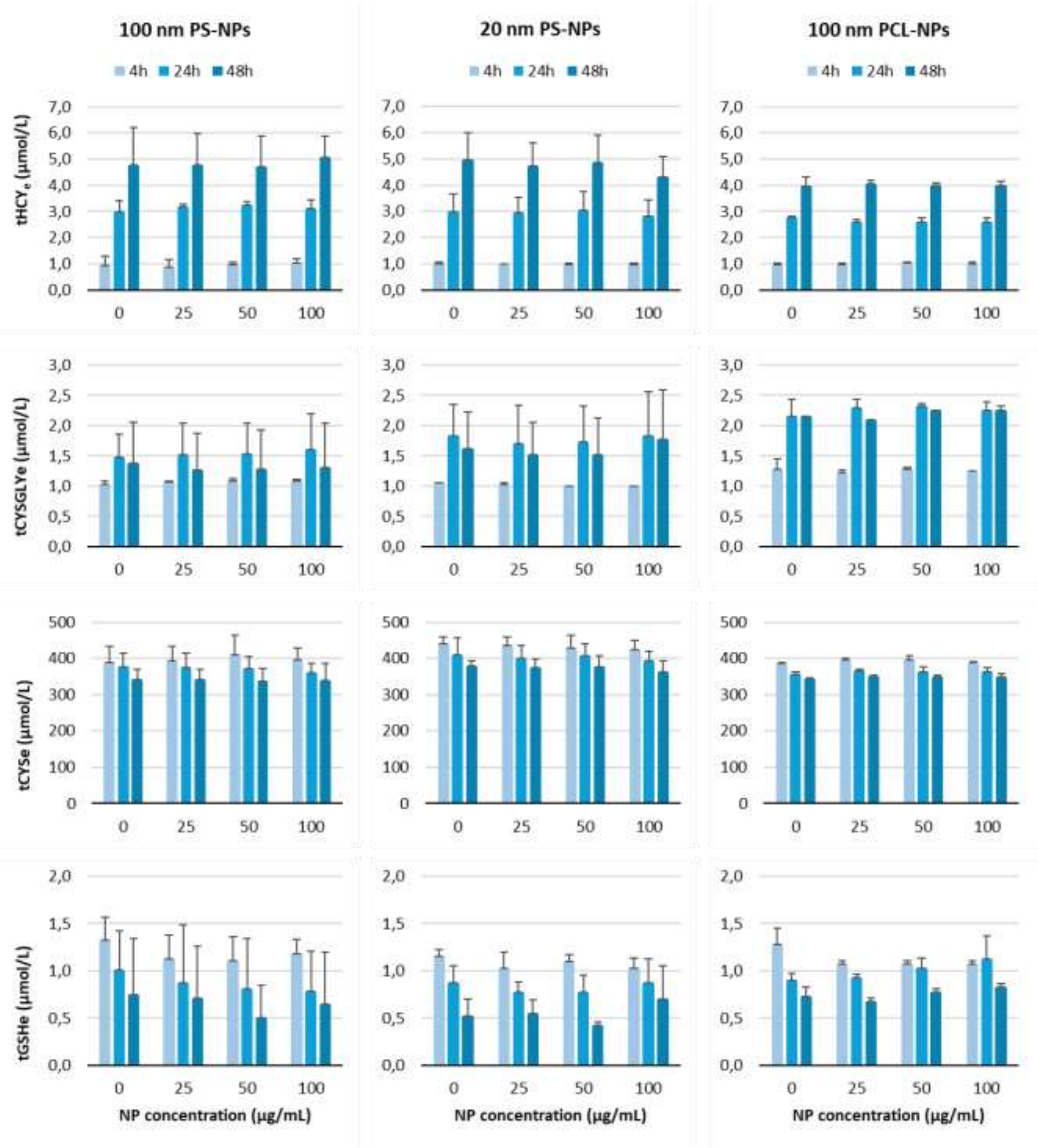

Figure S2: Glutathione and related metabolite concentrations in Caco-2 cell culture medium following exposure to 100 nm PS-NPs, 20 nm PS-NPs, and 100 nm PCL-NPs for 4, 24, and 48 hours. Data are mean ± SD. Two-way ANOVA showed a significant time effect ($p < 0.05$), with no effect of NP treatment.

Comparison of Body bends alterations in P0-F2 after exposure to PS-NPs

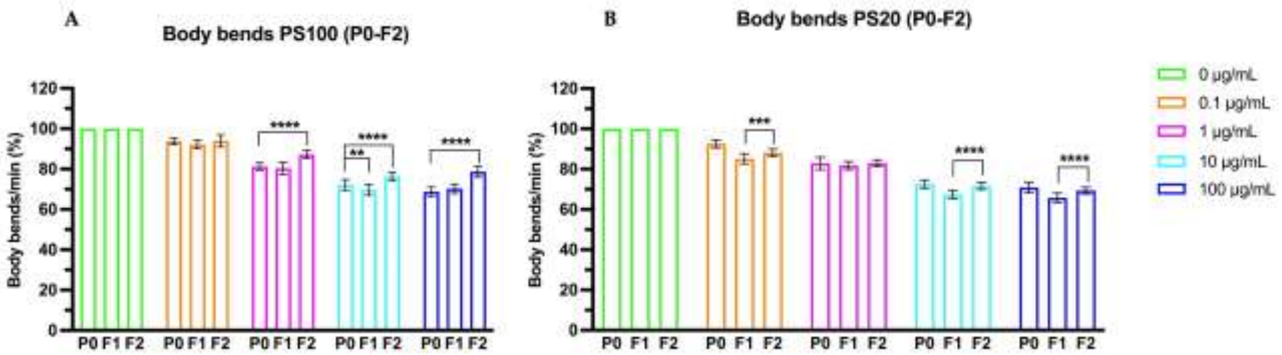

Figure S3: Comparison of body bends (P0–F2) following exposure to 100 nm PS-NPs (A) and 20 nm PS-NPs (B). Data are presented as mean ± standard deviation (SD) ** p<0.05; ****p<0.0001 (Ordinary two-way ANOVA with Bonferroni's multiple comparison tests, with a single pooled variance)

Comparison of Oxidative stress response in P0-F2 after exposure to PS-NPs

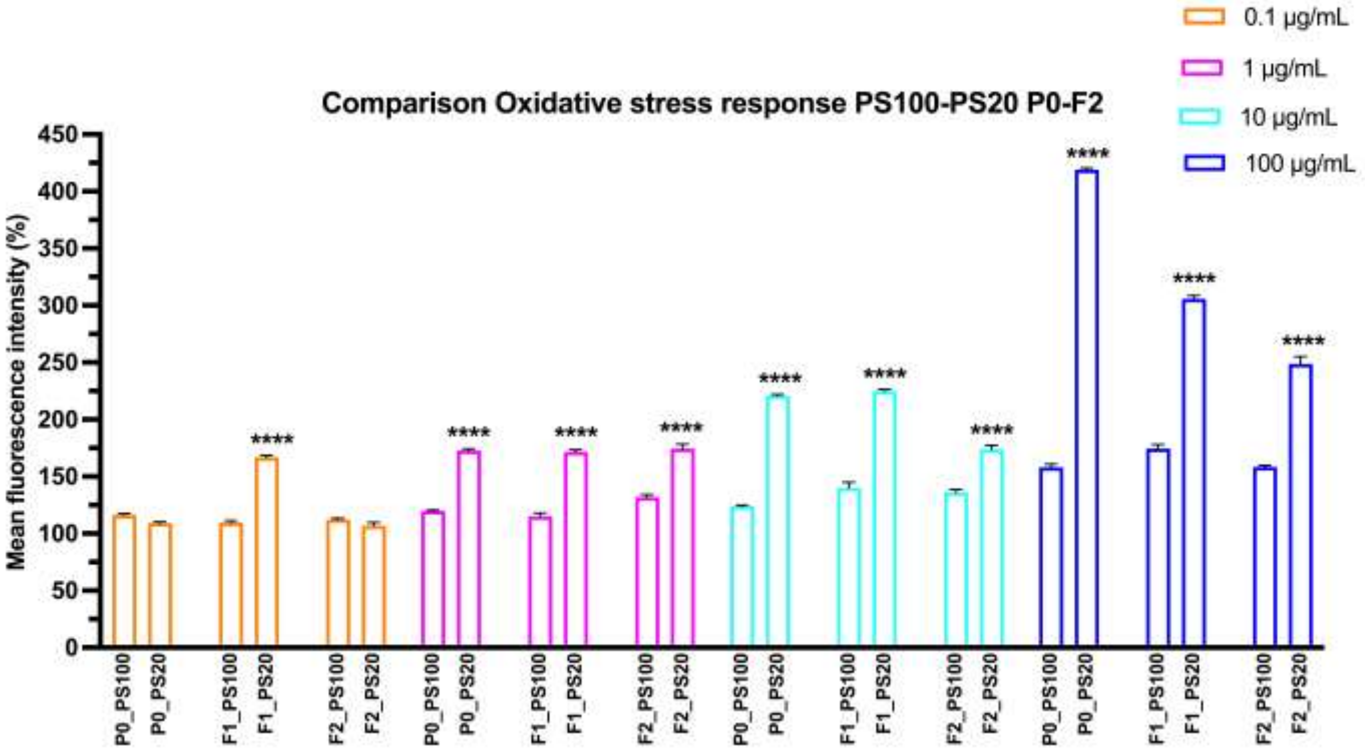

Figure S4: Comparison of the effects of exposure to different concentrations of 100 nm and 20 nm PS-NPs on the oxidative stress response of C. elegans across generations P0–F2. Data are presented as mean ± standard deviation (SD), normalized to control group (0 µg/mL=100%). ****p<0.0001 (Ordinary two-way ANOVA with Bonferroni's multiple comparison tests, with a single pooled variance)

Cell culture

Caco-2 and HT29-MTX cells were cultured in DMEM high glucose (Dulbecco's modified Eagle medium) supplemented with 10% heat-inactivated Fetal Bovine Serum (FBS), 1% non-essential amino acids, 1% L-glutamine and 1% penicillin/streptomycin. Raji B cells were cultured in suspension in RPMI high glucose supplemented with 10% heat-inactivated FBS, and 1% Penicillin/Streptomycin. All the reagents used for the cultures were purchased from Gibco (Gibco-Thermo Fisher Scientific, Waltham, MA, USA).

Briefly, Caco-2 and HT29-MTX cells were seeded at a 9:1 ratio into Polyethylene Terephthalate Transwell® inserts (3 µm pore size, Millipore®) placed in 12-well plates. On day 14 of

Caco-2/HT29-MTX co-culture 5 × 10^5 Raji B cells were added to the basolateral compartment to induce M phenotype in Caco-2 cells. Raji B cells were removed and reseeded on days 16 and 19. Co-cultures were maintained for 21 days to obtain a well-differentiated intestinal barrier.

Cytotoxicity

The NPs cytotoxicity was assessed using the 3-(4,5-dimethylthiazol-2-yl)-5-(3-carboxymethoxyphenyl)-2-(4-sulfophenyl)-2H-tetrazolium (MTS) cell viability assay, through the CellTiter 96® AQueous One Solution Cell Proliferation Assay (Promega Corporation, Madison, WI, USA). It was performed, on Caco-2 monolayer, according to the SOP developed in the EU NanoValid project (Nelissen et al., 2020, supplementary materials https://www.mdpi.com/2079-4991/10/8/1430/s1). The MTS assay measures cellular metabolic activity as an indicator of cell viability and cytotoxicity, based on the mitochondrial enzymatic reduction of the MTS tetrazolium compound to a soluble formazan product in metabolically active cells. Briefly, Caco-2 cells were cultured into 96 well culture plate After 24 hours, cells were treated with fivedifferent NPs concentrations of 1, 10, 25, 50, and 100 µg/ml for 24 and 48 hours. Benzalkonium chloride (BC) (0–0.05mg/ml) was used as a positive control. At the end of incubation time, treatments media were removed, cell monolayers were carefully washed with phosphate-buffered saline (PBS) and 150µl of MTS solution was added to each well. After 3 hours, formazan absorbance was measured at 490 nm by microtiterplate reader (Nivo, Perkin Elmer, Waltham, MA, US).

Comet Assay

DNA damage, as double and single strand breaks, was evaluated by alkaline comet assay. Caco-2 cells were seeded in 12-well cell culture plates. Briefly, cells were embedded in 0.7% low melting agarose (LMA, Bio-Rad Laboratories, Hercules, CA, USA) and put on 1% normal melting agarose (NMA, Bio-Rad Laboratories, Hercules, CA, USA) precoated slides. After that, slides were immersed in cold lysis solution (100-mM Na_2EDTA, 2.5-M NaCl, 10-mM Tris-HCl, 10% DMSO e 1% Triton X, pH 10) overnight at 4 °C. Afterwards, slides were transferred in alkaline buffer (300-mM NaOH, 1-mM Na_2EDTAat pH 13) for 20 min, at 4 °C. Electrophoresis was carried out for 30 min at 0.8 V/cm and 4°C. Subsequently, slides were neutralized with 0.4-M Tris–HCl (pH 7.5) buffer, fixated in cold absolute ethanol and stained with Ethidium Bromide (Merck/Sigma-Aldrich St. Louis, MO, USA). Slides were analyzed using a fluorescence microscope (Leica, Wetzlar, Germany) equipped with an image analysis system (IAS 2000 Delta Sistemi Srl) to provide various quantitative DNA damage parameters at single cell level. Tail intensity, intended as the percentage of DNA in the comet tail was selected as the most suitable parameter for evaluating DNA damage. The enzyme-modified version of comet assay was performed according to that developed by Comet Assay Interest Group (Collins et al., 1996). After lysis, slides were incubated with 1-µM formamidopyrimidine DNA-Glycosylase (FPG) (Merck/Sigma-Aldrich St. Louis, MO, USA) in 1X enzyme reaction buffer (40-mM HEPES, 0.1-M KCl, 0.5-mM EDTA, 0.2-mg/ml bovine serum albumin (BSA), and pH 8.0 with KOH) and incubated at 37 °C for 30 min. At the end of incubation, slides were immersed for 40 min in alkaline buffer and then undergone to the same procedure of the standard protocol, described above. All the experiments were performed in triplicate for each sample, and results reported as mean± standard deviation (SD) of tail intensity values.

Barrier integrity

Trans-epithelial Electrical Resistance (TEER) was assessed to determine the integrity of the *in vitro* intestinal barrier model, on day 14 of Caco-2/HT29-MTX co-culture and on day 21 of Caco-2/HT29-MTX/Raji B tri-culture, before performing the experiments. TEER was evaluated by a chop-stick electrode device (Millicell ERSVoltameter-Millipore-Sigma, San Luis, Mo, USA). Three separate measures were performed for each insert. Results were expressed as ohms × cm^2 according to the following formula (1):
TEER = [Ω cell monolayer − Ω filter (cell-free)] × filter area (1.12 cm^2). The inserts are considered suitable for the experiments if TEER values are above 150 Ω cm^2).

Paracellular permeability

Briefly, after treatment removal from the Ap compartments of the inserts and Ap and Bl chambers were washed twice with Hanks Balanced salt solution Buffer (HBSS). Then 0.4 mg/mL of Lucifer Yellow (Merck/Sigma-Aldrich St. Louis, MO, USA) in 0.5 mL of HBSS were added in the Ap compartment, while the Bl side was filled with 1 mL of HBSS. After 2 hours of incubation at 37 °C, 100 µL of the Bl volume from each insert was collected and transferred into a black microtiter 96 well-plate (Perkin Elmer, Waltham, MA, US). Samples were analysed by spectro-fluorimetry (Nivo, Perkin Elmer, Waltham, MA, US) at λ 485–528 for LY. Results were expressed as Papp according to the following formula (2):

Papp = ((DQ/Dt) × V) × (1/AC0) (2) where DQ/Dt is the amount of LY transported in the Bl compartment per time unit (t), V is the Bl volume (cm^3), A is the surface area of the filter (1.12 cm^2) and C0 is the initial concentration in the Ap compartment.

Oxidative stress

At the end of the cell treatment, the extracellular medium was removed and stored at −80 °C. Cells were then washed twice with PBS, and 0.2 mL of fresh PBS was added to each well before freezing the plate at −80 °C. On the day of the assay, cells were thawed and homogenized by placing the plate in an ice–water ultrasonic bath for 10 min (Ultrasonik 104H, NeyTech, USA). Subsequently, cell remnants were carefully scraped while keeping the plate on ice, and the whole-cell lysate was transferred into 1.5 mL tubes. A 100 µL aliquot of the lysate was then transferred into a new tube containing an equal volume of ice-cold trichloroacetic acid solution (100 g/L) with 1 mM EDTA. After 5 min on ice, acidified samples were centrifuged (13,000 × g, 5 min, 4 °C), and the supernatants were immediately used for the analysis of $rGSH_i$, while an aliquot was stored at −80 °C for the determination of $tGSH_i$. Non-acidified cell lysates were used on the same day for protein quantification using the Bradford assay.
For the determination of $tGSH_i$, 15 µL of supernatant from the acidified cell lysate were mixed with 15 µL of 0.3 M NaOH, 70 µL of internal standard (6 µM cystamine dihydrochloride), and 10 µL of a 100 g/L tris(2-carboxyethyl)phosphine (TCEP) aqueous solution. The mixture was incubated for 10 min at 37 °C (reduction step). Subsequently, 90 µL of PBS were added, and 50 µL of this mixture were transferred into a new tube containing 125 µL of 0.125 M borate buffer with 4 mM EDTA (pH 9.5), 6 µL of 0.3 M NaOH, and 50 µL of 1 g/L 7-fluorobenzofurazan-4-sulfonic acid (SBD-F, ammonium salt) in 0.125 M borate buffer. Samples were incubated for 60 min at 60 °C (derivatization step), filtered through a 0.22 µm RC membrane filter, and transferred into autosampler vials for HPLC analysis.

For the determination of extracellular thiols, 150 µL of culture medium were mixed with 50 µL of internal standard and 20 µL of TCEP solution, and the mixture was incubated for 10 min at 37 °C (reduction step). Subsequently, 200 µL of cold TCA/EDTA solution were added to each sample, and the samples were kept on ice for 5 min before centrifugation (13,000 × g, 5 min, 4 °C). Then, 150 µL of the supernatant were mixed with 15 µL of 3.5 M NaOH, 25 µL of 0.625 M borate buffer containing 20 mM EDTA (pH 9.5), and 50 µL of 1 g/L 7-fluorobenzofurazan-4-sulfonic acid (SBD-F, ammonium salt) in 0.125 M borate buffer. The subsequent steps of the assay were performed as described for the intracellular GSH determination.

Thiol separation was performed by injecting 40 µL (10 µL for extracellular thiol analysis) of the derivatized sample into a C18 column (Luna Omega C18 100 Å, 150 × 4.6 mm, 5 µm particle size; Phenomenex), protected by an ODS guard cartridge (10 × 4.6 mm; Supelco). The mobile phase consisted of 0.1 M phosphate buffer (pH 2.1) containing 35 mL/L acetonitrile. Chromatographic runs were carried out isocratically at a flow rate of 1 mL/min and a constant temperature of 28 °C. The HPLC system included a pump unit with degasser, an autosampler, and a column oven (Flexar, PerkinElmer), a fluorescence detector (2475 Multi λ Fluorescence Detector, Waters), and a PC-driven data acquisition module (NCI-901, PerkinElmer). SBD-derivatized thiols were detected at 385 nm excitation and 515 nm emission wavelengths. Chromatograms were processed using the TotalChrom Workstation software (version 6.3.2, PerkinElmer).

Standard solutions containing appropriate concentrations of the oxidized forms of HCY, CYS, CYS–GLY, and GSH were freshly prepared in PBS or in cell culture medium and analyzed in the same way as the samples. Thiol concentrations were calculated using a linear calibration curve obtained from the ratios of thiol to internal standard peak areas.

***C. elgans* exposure study design**

The exposure scenario of NPs was designed to mimic long-term human exposure. For pre-treatment, a mixture of *E. coli* OP50 (10^{10} colony forming units [CFU]/mL) and MNPs were evenly spread onto NGM plates and air-dried. The mixture was renewed daily throughout the exposure period. Stock solutions (100mg/ml for PS-NPs 100 nm and 10 mg/mL for PS-NPs 20 nm and PCL-NPs) were first diluted in ddH_2O to a working concentration of 5mg/ml. From these working solutions, the test concentrations (0-100 µg/mL) were prepared. The PS-NPs concentrations were selected based on a previous study (Yu et al., 2021), which showed that 10, 50, and 100 mg/L PS-NPs induce toxicity in *C. elegans*. Synchronized L1 larvae, obtained using the *C. elegans* Synchronizer System CES-700 (https://www.nemasync.com), were incubated with or without NPs at the concentrations indicated above for three days at 20 °C, representing long-term exposure (Wang, 2020). The worms were then allowed to grow to adulthood and subsequently prepared for the various assays. Exposure procedures were carried out up to the F2 generation.
